# A kinetic model for Pd-based hydrogenation of acetylene-rich streams typical of post-plasma applications

**DOI:** 10.1039/d5cy00529a

**Published:** 2025-09-03

**Authors:** Victor Rosa, Fabio Cameli, Yves Schuurman, Kevin M. Van Geem, Georgios D. Stefanidis

**Affiliations:** a Laboratory for Chemical Technology, Ghent University Tech Lane Ghent Science Park 125 B-9052 Gent Belgium kevin.vangeem@ugent.be; b School of Chemical Engineering, National Technical University of Athens Iroon Polytechniou 9 15780 Athens Greece gstefani@mail.ntua.gr; c IRCELYON, Institut de Recherches sur la Catalyse et l'Environnement de Lyon, UMR5256 CNRS-Universit'e de Lyon 69626 Villeurbanne France

## Abstract

The advancement of electrified chemical processes prompts interest in novel technologies such as plasma-based methane (CH_4_) conversion into high-demand chemicals. Specifically, nanosecond-pulsed discharges (NPDs) coupled with downstream Pd-based catalysts have demonstrated the best performance in a two-step, integrated process for converting CH_4_ into ethylene (C_2_H_4_). Given the untested composition range involved in this application, the focus of this work is the isolated performance of Pd-based catalysts in typical post-plasma conditions. Extensive campaigns of experiments are run in both traditional and novel stream compositions. The differences with traditional tail-end olefin-rich hydrogenation are highlighted, and a hybrid steady-state kinetic model is proposed, combining the traditional Langmuir–Hinshelwood–Hougen–Watson (LHHW) approach with an improved reversible adsorption methodology. The ability to accurately predict C_2_H_2_ hydrogenation kinetics with C_2_H_2_-rich and C_2_H_4_-poor streams is achieved by the new model, contrary to existing conventional models. Preliminary insights into catalyst optimization for scalable plasma-to-olefin routes are presented.

## Abstract 2

The advancement of electrified chemical processes prompts interest in novel technologies such as plasma-based methane (CH_4_) conversion into high-demand chemicals.

## Graphical abstract

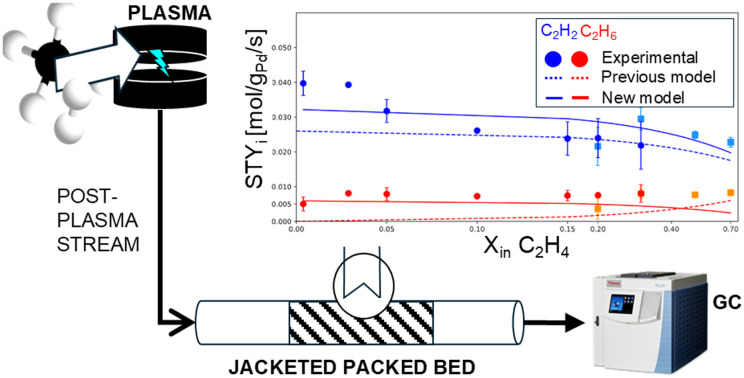

## 1. Introduction

The chemical industry is continually evolving, making the development of novel and sustainable chemical pathways increasingly critical. Electrified chemical processes are paving the way to the EU's goal of carbon neutrality by 2050, attracting widespread attention from academic research to industrial innovation. One promising approach in this landscape involves the conversion of hydrocarbon streams to commodity chemicals, such as ethylene (C_2_H_4_), through plasma technology. The electrified production of C_2_H_4_ from methane (CH_4_) has both the potential to utilise economically stranded sources of natural gas^42^ and to fit the needs of olefin producers, whose efforts to electrify steam cracking will require a sustainable route for CH_4_ byproduct utilization.^1^

In this context, the use of plasma technology represents an interesting method for valorising hydrocarbon streams. By igniting a discharge between two electrodes, plasma can break and rearrange C–C and C–H bonds, creating unsaturated, higher-value compounds from linear alkanes using electricity. Both thermal arc plasmas, where high temperatures (up to 10^4^ K) drive reactivity, and non-thermal applications, where high-energy electrons contribute to chemical reactions, have been successfully demonstrated at various scales.^2,3^

Acetylene (C_2_H_2_)-rich hydrogen (H_2_)–CH_4_ mixtures are the product of plasma-based reactors starting from CH_4_-based feedstocks, whose typical inlet/outlet compositions are reported for the most used discharge technologies in Table 1. Their limited selectivity towards higher-demand C_2_ building blocks like C_2_H_4_ remains one of the main obstacles to scalability,^3^ next to the inherent technological complexity of plasma reactors. Recent trials have explored the coupling of nanosecond-pulsed discharge (NPD) plasma reactors with downstream catalytic hydrogenation, achieving high single-pass yields of C_2_H_4_ from CH_4_ at a small scale.^4,5^ Seemingly, this approach would solve the aforementioned selectivity issues. However, the feasibility of similar plasma-to-olefin routes is closely tied to a better understanding of catalytic behaviour in post-plasma conditions, a topic largely unexplored in existing literature. Owing to the projected rise in C_2_H_4_ demand, addressing this issue will become ever more crucial.

**Table 1:** Summary of main technologies adopted in C_2_H_2_ and C_2_H_4_ production from CH_4_*via* plasma, and comparison with conventional steam cracking tail-end hydrogenation conditions

Technology	Feedstock composition (mol mol^−1^)	Hydrogenation inlet stream composition (mol mol^−1^)
Thermal arc + fast quenching^6^	CH_4_	C_2_H_2_ (0.25), H_2_ (0.75) with instant quenching, C_2_,C_3_^+^ coke otherwise
Nanosecond-pulsed plasma (NPD)^5^	CH_4_ (0.5), H_2_ (0.5)	C_2_H_2_ (0.07), C_2_H_4_ (0.005), CH_4_ (0.2), H_2_ (0.67), coke
Dielectric-barrier-discharge (DBD)^7^	CH_4_ (0.25), H_2_ (0.25), He (0.5)	C_2_H_6_ (0.01), CH_4_ (0.23), H_2_ (0.26), He (0.50), minor C_3_ products
Gliding/rotating arc (GDA)^8^	CH_4_	C_2_H_2_ (0.1), H_2_ (0.175), CH_4_ (0.5), coke
Tail-end hydrogenation in steam cracking^9–11^	Naphtha, propane (C_3_H_8_), ethane (C_2_H_6_)	C_2_H_2_ (0.01), C_2_H_4_ (0.8), H_2_ makeup

In large-scale industrial hydrogenation, widely performed in a tail-end configuration as part of the steam cracking process, traces of C_2_H_2_ (0.005–0.02 mol mol^−1^) are removed from already purified C_2_H_4_-rich streams using a low wt% Pd/Al_2_O_3_ catalyst (0.015–0.05 wt%, Table 1).^10,12,13^ By contrast, post-plasma hydrogenation involves C_2_H_4_-poor streams with substantially higher amounts of C_2_H_2_, *e.g.* 0.07–0.09 mol mol^−1^, using high wt% Pd/Al_2_O_3_ catalyst (1 wt%).^4,5,14^ It is well known that metal loading affects metal nanoparticle size and dispersion, and that the effect of the latter on conversion and selectivity in C_2_H_2_ hydrogenation is not negligible.^15,16^ The differing catalysts and streams used between traditional, industrial conditions and new post-plasma applications require an extension of existing kinetic models.

**Fig. 1:**
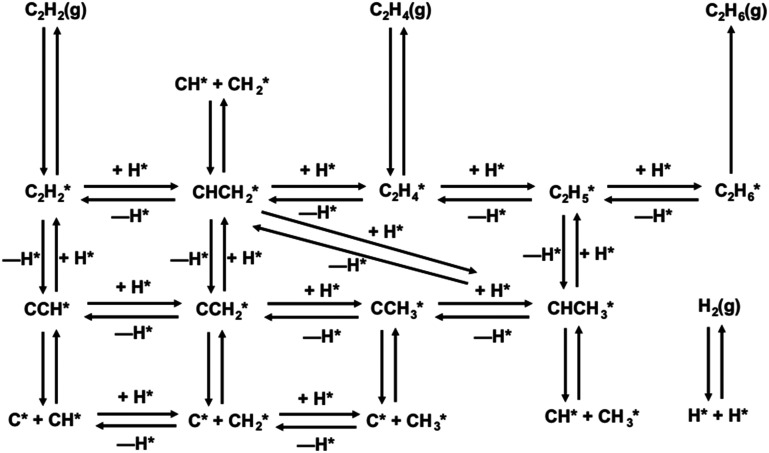
Overall reaction network of C_2_H_2_ hydrogenation on Pd. Reproduced from literature^30^ with permission. It includes adsorption reactions, desorption reactions, the main surface reactions and secondary surface reactions.

The large amount of literature regarding the reaction mechanism of C_2_H_2_ hydrogenation on Pd agrees upon the Horiuti–Polanyi nature of the surface mechanism^11,17–20^ (Fig. 1). Competitive adsorption of C_2_H_2_ and hydrogen (H_2_) is followed by step-wise H_2_ migration steps, concluded by desorption of gas-phase C_2_H_4_ and associative desorption of ethane (C_2_H_6_), passing through the vinyl (CH_2_=CH), π- or σ-adsorbed C_2_H_4_ and ethyl (CH_3_–CH_2_) surface intermediates, respectively. The presence of a wide range of secondary adsorbates such as ethylidene (CH_3_–CH), vinylidene (CH_2_=CH) and ethylidyne (CH_3_–C) has been ascertained through a variety of computational and experimental studies, with varying degrees of postulated roles in the mechanism.^17,21–25^ Several works have investigated the prediction of single steps' activation barriers *via* quantum chemistry, both on primary and secondary reaction paths.^18,19,26–28^ Conversely, only simpler Langmuir–Hinshelwood–Hougen–Watson (LHHW)-type models have been validated and or fitted on realistic sets of experimental data.^11,13,18,29^ Given the growing demand for detailed (micro)kinetic models with the ability to describe wide ranges of catalysts/operating conditions, the preliminary validation of a simplified hydrogenation kinetic model for C_2_H_2_-rich, C_2_H_4_-poor feedstocks (*i.e.* post-plasma conditions) is highly beneficial.

It is the goal of this work to show that a hybrid approach between a traditional LHHW-type model and a microkinetic methodology employing a reversible adsorption hypothesis can provide the best prediction of Pd-based C_2_H_2_ hydrogenation in a post-plasma regime of interest. A methodology leading to an exact analytical solution is proposed with a computational cost equivalent to a LHHW/power-law model, providing a higher level of description. Tail-end hydrogenation literature data^11^ are expanded with new experimental data in a wider gas composition range using an industrial surrogate 0.05 wt% Pd/Al_2_O_3_ catalyst. Post-plasma representative data with variable C_2_H_2_/C_2_H_4_ ratios streams and a 1% wt Pd/Al_2_O_3_ catalyst is gathered in a jacketed, cooled reactor. Overall, the proposed kinetic model, achieves the best predictive performance to date after regression in different operating conditions, offering preliminary insights into the design of post-plasma reactors.

## 2. Materials and methods

### 2.1 Experimental and kinetic details

#### 2.1.1 Catalyst preparation

The α-Al_2_O_3_-supported Pd catalyst beads are synthesized *via* incipient wetness impregnation. Various solutions of Pd(NO_3_)_2_ (Alpha Aesar) are prepared and added to α-Al_2_O_3_ powder (Alpha Aesar, 99.95% purity, particle size: 0.25–0.45 μm, pore volume: 0.35 mL g^−1^), which is sieved to attain a particle size between 100 and 200 μm. Details of the wt% of Pd and particle size of each catalyst employed in the experimental campaign are reported in the following sections. The impregnated catalysts are calcined at 600 °C in air for 6 h.

#### 2.1.2 Catalyst characterization

The active wt% of Pd in the two catalysts used, *i.e.* 0.05 wt% Pd/α-Al_2_O_3_ and 1 wt% Pd/α-Al_2_O_3_, is confirmed *via* inductively-coupled plasma (ICP) characterization. The active metal dispersion is measured *via* repeated H_2_ chemisorption experiments, leading to an average value of 38% for the 0.05 wt% Pd/α-Al_2_O_3_ catalyst and 10% for the 1 wt% Pd/α-Al_2_O_3_ catalyst.

#### 2.1.3 Packed bed experimental setup

**Fig. 2:**
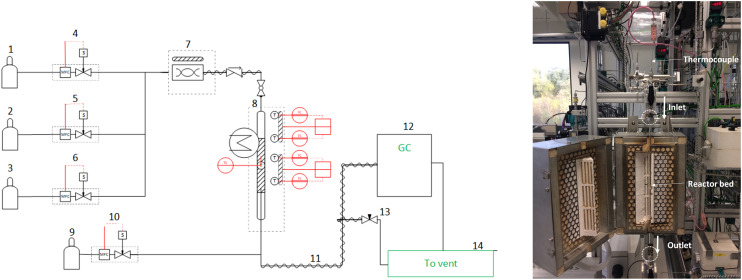
Left) Schematic overview of the catalytic reactor setup used for experiments. 1–3) Hydrocarbon mixture gas cylinders (at nominal pressures of 10–50 bar). 4–6) Calibrated Bronkhorst mass flow controllers (MFCs). 7) Evaporator. 8) Jacketed catalytic reactor and surrounding furnace, with multiple thermocouple-mediated control points for temperature. Three thermocouples are placed in the surrounding furnace and one is placed directly inside the catalytic bed, all shown in red colour. 9) He internal standard gas cylinder. 10) MFC controlling the internal standard fed to the reactor outlet line. 11) Heated reactor outlet line. 12) GC analysis of reactor effluent stream. 13) Needle valve controlling indirectly the vented fraction of the reactor effluent stream. 14) Vent section of the setup (details such as condensers are omitted for simplicity). Right) Picture of the setup used for tail-end and post-plasma reactive experiments.

A 6.5 mm internal diameter glass quartz reactor packed with a catalytic bed, is used for all experiments of interest in a temperature-controlled environment. The use of a cooling jacket with a volatile liquid coolant (ethanol, acetone) is adopted in highly exothermal regimes (adiabatic temperature rise >100 K). The reactive setup and the placement of the reactor quartz tube are shown in Fig. 2, highlighting the inlet and outlet sections of the reactor, the analytical section as well as the small diameter of the tube employed. The catalyst bed height is measured under 10 mm in all of the experimental runs. The absence of intra-/extra-particle mass- and heat-transfer limitations is ascertained through the EUROKIN tool.^31,32^

Catalyst pellet size, dilution material, gas velocity, tube diameter, material and thickness and observed reaction rates are used as input values together with the reactant's physical properties to identify an operational range which excludes aforementioned transport limitations. The thermal dilution material is used in the calculation of average bed properties (*e.g.* density, solid thermal conductivity) owing to its larger proportion (more in the next sections). Each experimental point is considered isothermal at the temperature measured by the internal reactor bed thermocouple, and the pressure is assumed uniform across the bed length owing to the limited pressure drop observed (max. 0.4–0.6 bar). A fixed value of bed porosity equal to 0.35 is adopted for all cases according to a well-known semi-empirical relation from literature,^32^ where ratios of tube diameter and particle diameter above 20 lead to an asymptotic value of porosity (*i.e.* 0.35). Product gases (up to C_4_ hydrocarbons) are detected *via* an online Gas Chromatograph (GC, ThermoFisher Scientific, Trace 1310) equipped with a thermal conductivity detector (TCD), a flame-ionization detector (FID), and a Molsieve 5A column and Hayesep-N column, using He as carrier gas. The calculated experimental values of C_2_H_2_ and C_2_H_6_ space–time yield (STY_*i*,exp_) are defined based on the following convention (eqn (1)):1
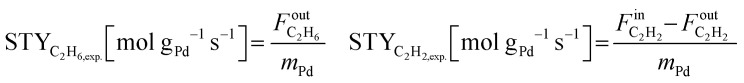
where 
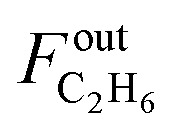
 is the outlet molar flowrate of C_2_H_6_ (mol s^−1^), 
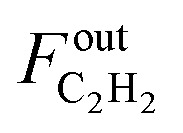
 is the outlet molar flowrate of C_2_H_2_ (mol s^−1^), *F*_C_2_H_2_,in_ is the inlet molar flowrate of C_2_H_2_ (mol s^−1^), and *m*_Pd_ (g) refers to the amount of (available) Pd present in the catalytic bed as measured from chemisorption. Details on the analytical calculation method for the outlet molar flowrates based on GC results, as well as on the calculation of the active amount of Pd are described in more detail in SI (sections S4 and S5).

Two operating scenarios are considered. The first involves an Ar-diluted hydrocarbon stream fed through the 0.05 wt% Pd/Al_2_O_3_ catalyst packed bed, representing an industrial-surrogate tail-end case. The second operating condition uses a highly reactive undiluted (or near-undiluted, max. 50%) hydrocarbon stream, fed through the 1 wt% Pd/Al_2_O_3_ packed bed representative of a CH_4_-fed post-plasma scenario. The two conditions are described in more detail in the next two sections.

#### 2.1.4 Diluted hydrocarbon stream experiments with a tail-end catalyst

In diluted experiments, 100 mg of a 0.05 wt% Pd/Al_2_O_3_ catalyst is sieved between 150 and 200 μm and thermally diluted with same-size Al_2_O_3_ in a ratio of catalyst/inert = 1/5. The tube is then placed inside an electric furnace to allow precise control of the temperature within the catalyst bed, achieved through K-type thermocouples whose location in the setup is highlighted in Fig. 2. All fresh samples are reduced in a 100 NmL s^−1^ flow of 1 : 1 H_2_ : Ar for 120 min at 200 °C, with a heating rate of 20 °C min^−1^. No additional reduction step is employed. Different gas mixtures are prepared and fed to the reactor combining the streams from the MFCs shown in Fig. 2, with a fixed total gas flowrate of 200 NmL s^−1^. Hereby, an extension of the C_2_H_4_ composition range is investigated in the 0–0.7 mol mol^−1^ inlet C_2_H_4_ molar fraction range, in light of its relevance in post-plasma stream compositions. The rest of the mixture's components are kept at a fixed inlet molar fraction of 0.007, 0.04, 0.019 mol mol^−1^ for C_2_H_2_, CH_4_, H_2_, respectively, with complementary Ar dilution to unity. No activation phase before steady-state achievement is observed. This procedure is analogous to that adopted by Urmès *et al.*,^11^ whose data is used as a comparative basis within this work. During the catalytic runs, the reactor is heated to compensate for heat losses and achieve isothermality at the desired reaction temperature, in the diluted conditions adopted. After experimentally reproducing the C_2_H_4_ rich data of Urmes (see section 3.1.1), we merge this work's data with their experiments in the variable inlet molar fraction range of C_2_H_4_, C_2_H_2_, H_2_, CH_4_, Ar = 0.25–0.9, 0.005–0.02, 0.01–0.065, 0–0.02, 0.2–0.7 mol mol^−1^. The combined data is later used within this work for model fitting purposes, providing the largest data set in variable reactant conditions for tail-end hydrogenation in literature.

#### 2.1.5 Undiluted hydrocarbon stream experiments with a post-plasma suitable catalyst

For undiluted stream experiments, 10 mg of a 1 wt% Pd/α-Al_2_O_3_ catalyst is sieved to a size between 80 and 100 μm to reduce intraparticle transport limitations in higher-volumetric-reaction-rate conditions (*i.e.* an undiluted case). The same catalyst has been previously used by the authors in a washcoated configuration for post-plasma hydrogenation,^4,5^ providing the only literature reference for an application of this kind. The powder form catalyst is hereby diluted with 500 mg of same-size SiC inert material, providing sufficient radial and axial heat transfer to avoid meaningful thermal gradients within the bed (max. 4 °C calculated within the EUROKIN tool). Analogously to diluted experiments, the bed is placed inside a quartz tube which is subsequently placed in an open furnace and cooled with an acetone-filled jacket through its latent heat of evaporation, effectively removing the exothermal heat of reaction. The low-conductivity glass quartz material represents the strongest heat transfer resistance in the reactive system, but its small thickness (1 mm) leads to minimal calculated effects on the space–time yields and provides stable operational points in steady-state conditions. The catalyst is pre-treated with the same procedure used for diluted experiments, and the same mass flow controllers are used to control the gas flows. The hydrocarbon streams are hereby probed in undiluted catalytic experiments with a variable 34–200 NmL min^−1^ flowrate and variable inlet molar fraction of C_2_H_4_, C_2_H_2_, H_2_, CH_4_ = 0–0.2, 0.02–0.1, 0.24–0.68, 0.1–0.25 mol mol^−1^, between 293 K and 343KC with no/close-to-no Ar dilution (0–50%, depending on the operating point). No activation phase before steady-state achievement is observed (see Fig. S3). A summary of the two different experimental conditions described in 2.1.4 and 2.1.5 is reported in Table 2.

**Table 2:** Grouped experimental conditions and catalysts investigated and/or used for model fitting within this work. The exposed Pd mass is calculated *via* H_2_ chemisorption (details in SI)

Condition	Catalyst	Catalyst mass	Exposed Pd mass	Dilution material	Temp. [°C]	Inlet fraction (mol mol^−1^)			
						C_2_H_2_	C_2_H_4_	H_2_	Ar
Diluted, tail-end	0.05 wt% Pd/α-Al_2_O_3_	100 mg	0.02 mg	Al_2_O_3_	51, 61	0.005–0.02	0–0.7	0.01–0.06	0.25–0.9
Undiluted, post-plasma representative	1 wt% Pd/α-Al_2_O_3_	10 mg	0.01 mg	SiC	30–80	0.02–0.09	0–0.2	0.48–0.68	0–0.5

#### 2.1.6 Kinetic assumptions and analytical expression

**Fig. 3:**
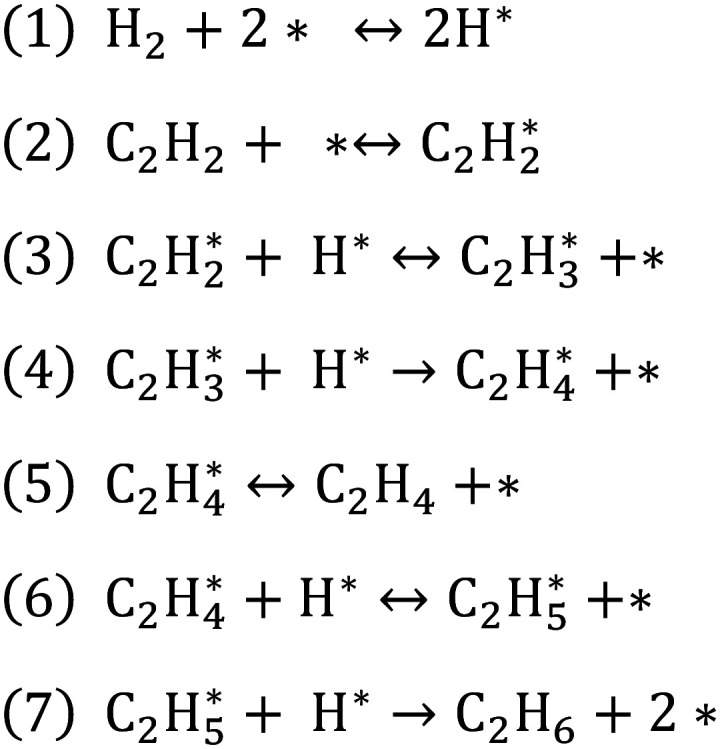
Simplified reaction scheme for C_2_H_2_ hydrogenation on a single Pd site.

A simplified, steady-state model is adopted to describe the hydrogenation kinetics of C_2_H_2_ and C_2_H_4_ on a single active Pd site. The required number of active sites for kinetic modelling purposes has been debated in the literature, and a two-active-site hypothesis has been proposed in some instances.^13^ Sufficient evidence is found to ascertain the validity of a single-site hypothesis in the conditions of interest, *i.e.* near-atmospheric pressure (1–2 bar) and absence of CO in the feed stream.^9,11,18,33^ The effect of CO is hereby neglected owing to the known effect of the latter on C_2_H_6_ selectivity and the kinetic complications that would result from it, *e.g.* invalidity of a single active Pd site assumption.^9,11^ The resulting condensed mechanism is shown in Fig. 3, following the main reaction path from acetylene to ethylene and through the vinyl and ethyl adsorbate intermediates, respectively. A range of secondary reactions involving vinyl, ethyl, ethylidene, ethylidyne, vinylidene adsorbates are hereby neglected, following the experimental observation that the latter's coverages are low compared to the main adsorbates considered in this work's mechanism (ethyl and vinyl).^17,21–25^ Carbide and hydride formation is also hereby neglected, owing to difficulties in their practical description and unclear quantitative effect on reactivity.^16,30^ The assumptions used to yield an analytical expression for the steady-state solution of the system are that steps 1, 2, 3, 6 are near-equilibrium and that steps 4 and 7 are irreversible, rate-determining steps (RDS). The RDS choice is motivated by a combination of prior evidence^19,27,34^ and strengthened by the data gathered in this work. Specifically, the hypothesis of step 1 (H_2_ adsorption) being rate-determining^18^ is rejected based on empirical evidence, further discussed in section 3 and SI (section S3). A meaningful difference to previous models is implemented in the description of C_2_H_4_ adsorption. Step 5 is assumed to be neither close to equilibrium, nor a rate-determining step, and is used in a reversible form -*i.e.* microkinetic approach- describing the C_2_H_4_ surface balance with two terms. The first term relates to the direct production of adsorbed C_2_H_4_*via* vinyl surface hydrogenation, and the second to its production *via* adsorption of gas-phase C_2_H_4_ (see SI, eqn. S13 and S14).

The overall consumption rate of gas-phase C_2_H_2_, *r*_C_2_H_2__, is assumed equal to the sum of production rates of gas-phase C_2_H_4_ and C_2_H_6_, *r*_C_2_H_4__ and *r*_C_2_H_6__, respectively. The latter implies neglection of secondary (parallel) reactions, as well as coupling/oligomerization reactions leading to C_4+_ species' production, justified by the near-complete carbon balances with this assumption in the conditions of interest (>85–90%, reported in SI Tables S3 and S4). The description of two out of three variables in the group *r*_C_2_H_2__, *r*_C_2_H_4__, *r*_C_2_H_6__ is used to close the mass balance in the system, as per eqn (2) and (3). Prior work has presented somewhat similar two-term expressions for *r*_C_2_H_6__,^13^ never capturing the correct dependence on all reactants' molar fractions (*i.e.* partial pressures) as will be shown throughout this work.2
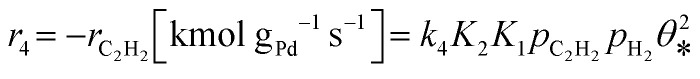
3
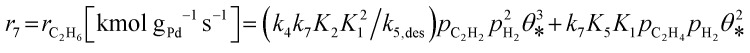
where *k*_4_, *k*_7_ are grouped rate constants in kmol g_Pd_^−1^ s^−1^, *K*_*i*_ is the equilibrium constant of the *i*-th step in kPa^−1^, *k*_5,des_ is the desorption rate constant of C_2_H_4_ in kmol g_Pd_^−1^ s^−1^, *p*_*i*_ is the partial pressure of the *i*-th gas-phase reactant measured in kPa, and θ_*_ is the fraction of free sites in monolayers (ML). The overall reaction rate expressions for C_2_H_2_ consumption and C_2_H_6_ production in eqn (2) and (3) contain two grouped constants, *k*_4_ and *k*_7_, which are the product between the equilibrium constants of two quasi-equilibrated steps (*i.e.* 3 and 6) and the forward rate constant of two irreversible steps (details in SI, eqn S21).

#### 2.1.7 Kinetic parameters

The grouped constants *k*_4_, *k*_7_ appearing in eqn (2) and (3) are described with a modified Arrhenius-type expression, arising from the product of the single reaction rate coefficients. The pre-exponential factor, temperature exponent and the activation energy are fitted with an average-temperature approach (illustrated in eqn (4)) according to prior methodology.^35^4
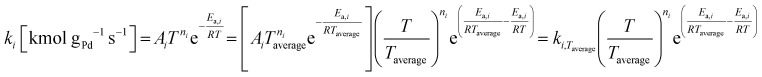
where *T* is the temperature of the single experimental measurement, *T*_average_ is the average temperature of the experimental set of measurements used for fitting, *A*_*i*_ is the pre-exponential factor (prefactor) for the grouped constant *k*_*i*_, *n*_*i*_ is its modified Arrhenius exponent, *E*_a,*i*_ is the activation energy of the latter, *R* is the universal gas constant expressed in [kJ mol^−1^ K^−1^], and *k*_*i*,*T*_average__ is the reaction rate constant calculated at the average reaction temperature. A value of *n*_*i*_ = 0 is used therein for diluted experiments (10 K temperature range), and a regressed value is used for undiluted experiments (50 K temperature range).

It is assumed that the temperature exponent involved in the modified Arrhenius expressions (eqn (4)) can account for the expectedly limited temperature dependence of the equilibrium constants, as well as the activation entropy of single steps. The activation energies *E*_a4_ and *E*_a7_ are assumed to be independent of coverage according to somewhat limited effects observed in prior studies^30^ and targeted simplicity of the model developed.

In accordance with prior work,^11,13^ a simplified approach incorporating fixed adsorption and desorption equilibrium constants is employed, defined as per eqn (5). The fitted values are compared with previous models and theoretical values in later parts of this work. Concurrently, the prediction of gas-phase equilibrium over a range of temperatures with established *ab initio* methodology^36^ is neglected for simplicity. The constant assumption is also used for the 
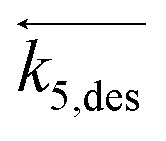
 value, neglecting its dependence on the C_2_H_4_ heat of adsorption to reduce parameter correlation (justified by the small temperature ranges used). Finally, the hypothesis of reversible C_2_H_4_ adsorption is used throughout this entire work to benchmark its predictive capabilities.

5 *K*_eq.*,i*_ [kPa^−1^] = constant

### 2.2 Reactor model and optimization algorithms

A one-dimensional, plug-flow pseudo-homogeneous reactor model is used to describe the reactive, catalytic behaviour of the hydrocarbon streams passing through the packed bed reactor. An intrinsic kinetic regime assumption is adopted in all experiments performed according to the details provided in section 2.1.3. The resulting system of ordinary differential equations (ODE) is solved in Python with the scipy.integrator.ode stiff ODE integrator, where the Cantera package is used to estimate all gas physical properties, such as mixture density and molecular weight (see SI, section S6). A value of 100 integration steps is used throughout this work, identified as the optimal compromise between accuracy and computational speed. Different Python instruments are used, with a combination of the gradient-based Levenberg–Marquardt (LM) algorithm implemented in the scipy.optimize.least_squares package and the Nelder–Mead (NM) optimization algorithm implemented in the scipy.optimize.minimize. The Nelder–Mead algorithm is a black-box unconstrained minimization tool often used in machine learning applications. When combined with the gradient-based methodology implemented in the LM algorithm, it outperforms true global optimization tools such as Bayesian optimization which have been tested in the problem of interest. Initialization of convergence far from rapidly diverging areas is achieved in this manner. Parameters are fitted by initial use of the NM algorithm followed by LM-based refinement. Evaluation of binary correlation parameters and 95% parameter confidence intervals is performed through the finite-difference approximation of the Jacobian matrix provided by the least_squares package, with a linear loss method. Such values are calculated and reported only for a subset of relevant parameters,^34^ despite having only approximate validity in a multiresponse nonlinear regression problem.^11,37^ Finally, the output of the model is probed in terms of modelled space–time yields (STY_*i*,model_) calculated as per eqn (6) and (7), as well as C_2_H_2_ conversion and C_2_H_6_ selectivity defined in eqn (8)6
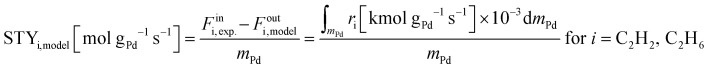
7
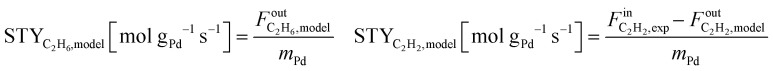
8
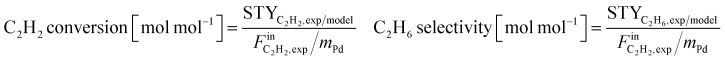
where *F*^out^_i,model_, *F*^in^_i,exp._ are the modelled outlet and the experimentally known inlet molar flowrate of reactant i, respectively, *m*_Pd,reactor_ is the mass of homogeneously distributed Pd in the catalyst bed, *r*_i_ is the instantaneous kinetic reaction rate for reactant i defined in section 2.1.6.

## 3. Results and discussion

The following sections (3.1 and 3.2) present a comprehensive analysis of the measured and modelled key performance indicators introduced in section 2.2, namely the STY of C_2_H_2_ and C_2_H_6_, under different operating conditions. Section 3.1 reports their experimental values, *i.e.* STY_C_2_H_2_,exp_ [mol g_Pd_^−1^ s^−1^] and STY_C_2_H_6_,exp_ [mol g_Pd_^−1^ s^−1^] calculated with on-line analytical instruments during reactive runs on the basis of eqn (1), and illustrates their dependence on manipulated variables. Section 3.2 benchmarks the corresponding model predictions, STY_C_2_H_2_,model_ [mol g_Pd_^−1^ s^−1^] and STY_C_2_H_6_,model_ [mol g_Pd_^−1^ s^−1^] calculated from the model output variables with eqn (9). Finally, conclusions are drawn on the validity of the fitted model parameters and their consistency based on the existing literature (section 3.3).

### 3.1 Experimental results

The experimental campaigns outlined in section 2.1.4, 2.1.5 are designed to describe the response of STY_C_2_H_2_,exp_ [mol g_Pd_^−1^ s^−1^] and STY_C_2_H_6_,exp_ [mol g_Pd_^−1^ s^−1^] to variations in reactant partial pressure and reactor temperature. This section is dedicated to the analysis of these results, in traditional (diluted) and post-plasma representative (undiluted) conditions that provide the reference sets for model validation in section 3.2.

#### 3.1.1 Diluted, tail-end hydrogenation experiments

The first operating scenario involves a tail-end catalyst fed with a diluted hydrocarbon mixture. The focus of these experiments is on a feed extension to C_2_H_4_-poor conditions, maintaining tail-end representative compositions of the other reactants (see diluted conditions in Table 2).

**Fig. 4:**
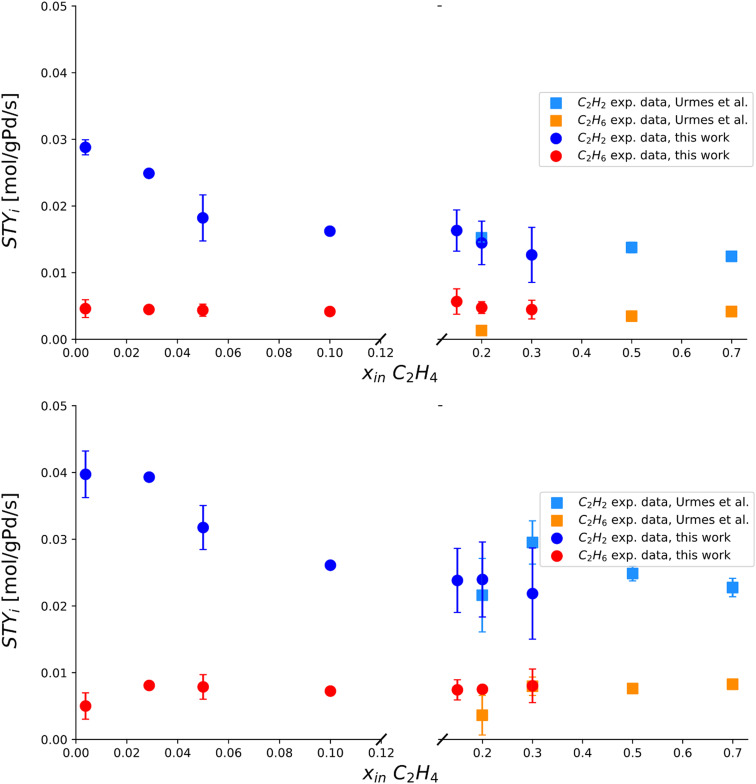
Experimentally measured space–time yields of C_2_H_2_ and C_2_H_6_ (STY_C_2_H_2__ [mol g_Pd_^−1^ s^−1^] and STY_C_2_H_6__ [mol g_Pd_^−1^ s^−1^]) plotted against the inlet molar fraction of C_2_H_4_. Top) Trend obtained at 323 K, *i.e.* 50 °C. Bottom) Trends obtained at 333 K, *i.e.* 60 °C. The *x*-axis is reported with a broken plot representation to precisely quantify the behavior across the composition range investigated. Includes experimental data by Urmes *et al.* and experimental data from this work. The standard deviation of experimental measurements is reported through error bars for points whose reproducibility has been verified through at least two repeated experiments.

The main results are shown in Fig. 4, which captures the transition zone from tail-end, C_2_H_4_-rich streams to C_2_H_4_-poor compositions with the aid of a broken plot with variable scale throughout the *x*-axis. An inverse proportionality between STY_C_2_H_2__ and inlet molar fraction of C_2_H_4_ is highlighted, in agreement with previous hypotheses. Conversely, a peculiar trend of STY_C_2_H_6__*vs.* C_2_H_4_ inlet molar fraction is disclosed, where a transition from a fully linear dependence on the inlet molar fraction of C_2_H_4_ (right side), to a weakly linear one (left side), is observed at inlet molar fraction = 0.2 mol mol^−1^. This leads to a non-zero value for the y intercept. Furthermore, the new experimental evidence is merged with recent tail-end data from Urmès *et al.*,^11^ with a color-aided representation of variables. The agreement between sets is verified at an inlet molar fraction of C_2_H_4_ = 0.2–0.3 mol mol^−1^ at the two different temperatures investigated (323–333 K), ensuring reproducibility with literature.

Finally, a marked sensitivity of space–time yields to temperature is observed in the limited 323–333 K range adopted, as expected from hydrogenation reactions whose start-of-run temperature in tail-end conditions can be as low as 313 K.^38^ More detailed analysis of thermal behaviour in tail-end conditions is beyond the scope of this work, which is rather the post-plasma condition investigated next.

#### 3.1.2 Undiluted, post-plasma hydrogenation experiments

**Fig. 5:**
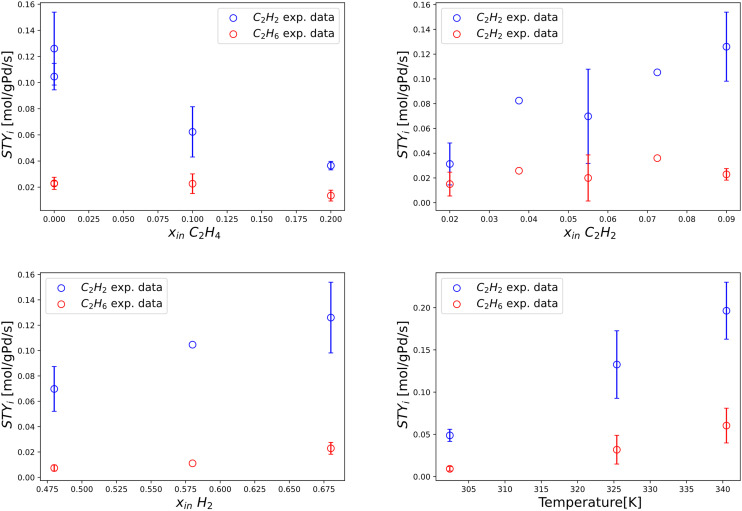
Experimentally measured space–time yields of C_2_H_2_ and C_2_H_6_ (STY_C_2_H_2__ [mol g_Pd_^−1^ s^−1^] and STY_C_2_H_6__ [mol g_Pd_^−1^ s^−1^]) plotted against single manipulated operating variables. Top left) Trends *vs.* inlet molar fraction of C_2_H_4_ obtained at 293 K, *i.e.* 20 °C. Top right) Trends *vs.* inlet molar fraction of C_2_H_2_ obtained at 293 K, *i.e.* 20 °C. Bottom left) Trends *vs.* inlet molar fraction of H_2_ obtained at 293 K, *i.e.* 20 °C. Bottom right) Trends *vs.* isothermal reactor temperature. The standard deviation of experimental measurements is reported through error bars for points whose reproducibility has been verified through at least two repeated experiments.

The results of a Pd-rich (1% wt) catalyst operated in post-plasma representative conditions (see Table 2) are presented in this section. The most relevant trends are reported in Fig. 5, disclosing a new operating range in literature. Four relations are investigated, regarding the dependence of STY_C_2_H_2__ and STY_C_2_H_6__ on the inlet molar fractions of C_2_H_4_, C_2_H_2_, H_2_ (at 303 K), and their isolated dependence on temperature at a fixed inlet molar composition of C_2_H_4_, C_2_H_2_, H_2_, CH_4_ = 0, 0.09, 0.68, 0.23 mol mol^−1^. The relatively wide error bars observed are explained by the complexity of maintaining a truly steady-state condition in the strongly exothermal regime investigated. Nonetheless, the jacketed reactor outlined in section 2.1.3 allows gathering the first evidence concerning Pd-based C_2_H_2_ hydrogenation in undiluted conditions where the adiabatic temperature rise is greater than 100 K.

In Fig. 5 (top left), the observed trend of STY_C_2_H_2__*vs.* inlet molar fraction of C_2_H_4_ shows a similar decreasing behaviour to diluted tail-end conditions, with an inverse relation between the two variables. The experimentally observed trend of STY_C_2_H_6__*vs.* inlet molar fraction of C_2_H_4_ is near-constant, analogously to diluted conditions. In Fig. 5 (top right), the trend of STY_C_2_H_2__*vs.* inlet molar fraction of C_2_H_2_ highlights direct proportionality in the left part of the plot with a possible maximum near the end of the trend. In turn, the dependence STY_C_2_H_6__ on inlet molar fraction of C_2_H_2_ is near-constant with an (apparent) maximum, albeit the width of error bars does not allow to make certain assessments concerning the presence of the latter. In Fig. 5 (bottom left), the trends of STY_C_2_H_2__ and STY_C_2_H_6__*vs.* inlet molar fraction of H_2_ are univocally increasing, as expected from a hydrogenation reaction. Finally, in Fig. 5 (bottom right), the temperature dependence of STY_C_2_H_2__ and STY_C_2_H_6__ is investigated in a post-plasma scenario, showcasing limited evolution to byproduct C_2_H_6_ in a larger range of temperature, where truly isothermal conditions can be achieved. The thermal behaviour of the catalyst is characterized for the first time in the transition phase from low-temperature points to post-plasma representative values, proving that relatively low amounts of C_2_H_6_ byproduct formation can be attained if the reaction heat is removed efficiently. The formation of hot spots thus represents the greatest challenge for future post-plasma applications, where a reactor with an elevated heat transfer coefficient can avoid such selectivity issues.

### 3.2 Regressed kinetic parameters and predictive efficacy

The kinetic regression routine outlined in section 2.3 is adopted to find the best-fit parameters in different conditions. 95% confidence intervals are reported in Table 3 for selected parameters in the two main scenarios investigated (*i.e.* diluted tail-end and undiluted post-plasma). The orders of magnitudes of *A*_4_, *A*_7_ present substantial variation among different conditions/sets. This is attributed to their coupling with activation energies *E*_a4_, *E*_a7_ and the respective exponential sensitivity to temperature. Similarly, the relatively wide confidence intervals observed for parameters in diluted conditions are attributed to the limited range of temperature (10 K) in which the exponential dependence of the Arrhenius expression is fitted. Owing to the exploratory nature of this work and the predictive accuracy of the model shown in the next sections, the validity of the model is deemed sufficient. Further analysis of the fitted adsorption constants follows in section 3.3.

**Table 3:** Set of experimentally fitted parameters and 95% selected confidence intervals in diluted tail-end conditions and undiluted post-plasma conditions with the model presented in section 2.1.6, as well as equilibrium parameters computed with transition-state theory and adsorption enthalpies from literature, with the methodology presented in SI

Value	*K*_1_	*K*_2_	*K*_5_	*k*_5,des_	*A*_4_	*A*_7_	*E*_a,4_	*E*_a,7_	*n*_1_	*n*_2_
This work, tail-end	4.3 × 10^3^	1.1 × 10^5^	2.4 × 10^2^	(2.5 ± 1.1) × 10^−4^	(2.4 ± 0.3) × 10^2^	28.5 ± 4.7	39.6 ± 11	33.3 ± 20.1	—	—
This work, post-plasma	2.9 × 10^4^	3.4 × 10^3^	2.5 × 10^3^	(3 ± 0.9) × 10^−3^	(4 ± 0.5) × 10^−3^	(1.7 ± 0.5) × 10^−3^	22.7 ± 0.8	27.0 ± 1.9	−0.02	0.09
Previous work, exp.^11,18^	10^4^	10^4^–10^5^	10–10^2^	Not defined	(1.1 ± 0.1) × 10^4^	(28.6 ± 0.5) × 10^5^	48.5 ± 4.2	54.8 ± 11	0	0
Previous work, *ab initio* estimate^19,25,28,39,40^	10^4^–10^5^	10^2^	10^3^–10^4^	Not defined						
Units	kPa^−1^			kmol g_Pd_^−1^ s^−1^			kJ mol^−1^		—	

#### 3.2.1 Model results in diluted, tail-end hydrogenation conditions

In this section, the predictions of the new model are probed on the combined experimental datasets from this work (C_2_H_4_-poor regime) and that of Urmès,^11^ in the widest range of tail-end compositions in which a C_2_H_2_ hydrogenation kinetic model has been fitted (to date). The operative conditions in which the model is employed, *i.e.* various C_2_H_2_, C_2_H_4_ and H_2_ inlet molar fractions, are summarized in Table 2. A portion of Urmes' data is incorporated into the model for fitting and validation purposes without additional experimental reproduction. Cross-reproducibility has been ascertained in a variable C_2_H_4_ range in section 3.1.1, justifying extension to other compositions from literature. Furthermore, tail-end conditions are only used to illustrate the model's ability to describe catalytic behaviour in a post-plasma representative composition (C_2_H_4_-poor), while maintaining general accuracy.

**Fig. 6:**
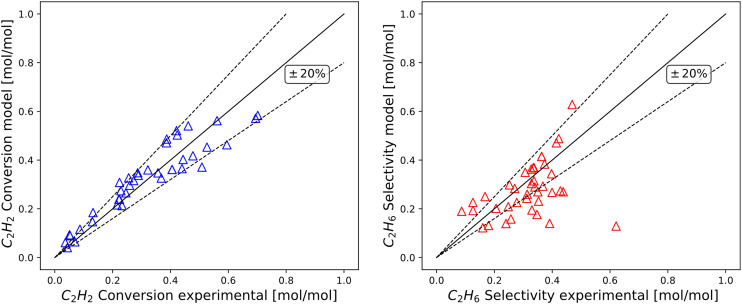
Parity plot for C_2_H_2_ hydrogenation prediction on a diluted hydrocarbon stream with ±20% relative deviation lines. The *y*-axis corresponds to the results of the computational model and *x*-axis follows experimentally obtained points. Left) C_2_H_2_ conversion right) C_2_H_6_ selectivity.

In Fig. 6, the experimental agreement of the model is introduced in terms of parity for C_2_H_2_ conversion and molar C_2_H_6_ selectivity, *i.e.* typical hydrogenation performance indicators. The limited dispersion highlights the efficacy of the model while using fixed values for equilibrium parameters *K*_1_, *K*_2_, *K*_5_ in close vicinity with recent work^11^ (see section 3.3 for deeper analysis).

**Fig. 7:**
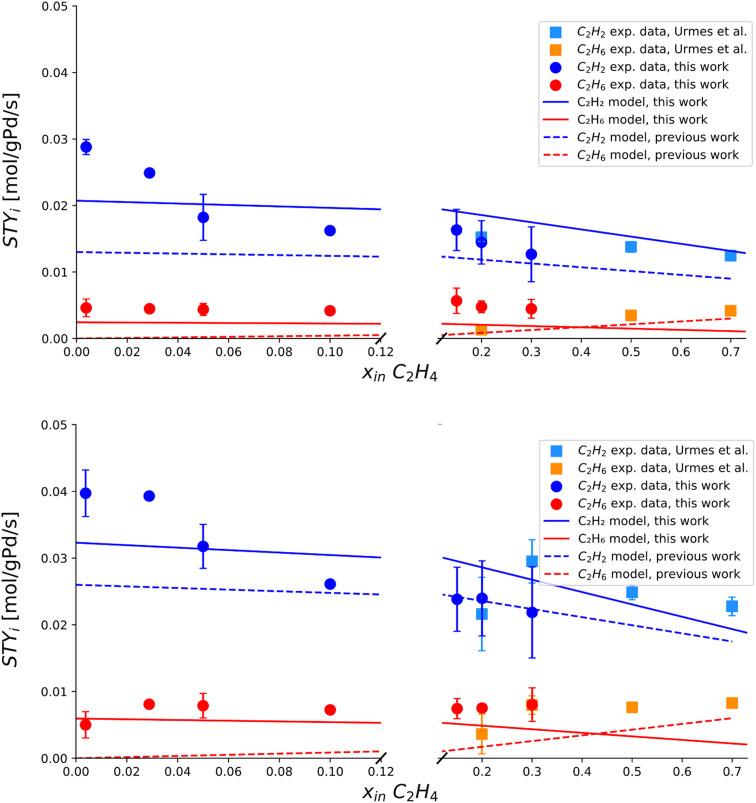
Experimentally measured and modelled space–time yields of C_2_H_2_ and C_2_H_6_ (STY_C_2_H_2__ [mol g_Pd_^−1^ s^−1^] and STY_C_2_H_6__ [mol g_Pd_^−1^ s^−1^]) plotted against the inlet molar fraction of C_2_H_4_. Top) Trend obtained at 323 K, *i.e.* 50 °C. Bottom) Trends obtained at 333 K, *i.e.* 60 °C. The *x*-axis is reported with a broken plot representation to precisely quantify the behavior across the composition range investigated. Includes experimental data by Urmes *et al.*, experimental data from this work, model simulation results by this work and a dotted line showing model simulation results by Urmès *et al.*^11^ The standard deviation of experimental measurements is reported through error bars for points whose reproducibility has been verified through at least two repeated experiments.

In Fig. 7, the predicted space–time yields are plotted against the inlet molar fractions of C_2_H_4_ in the widest (0–0.7 mol mol^−1^) range to date. Results from the new model are compared with the previous model of Urmès *et al.*,^9–11,41^ with color- and symbol-aided distinction. The trend of STY_C_2_H_6__*vs.* C_2_H_4_ inlet content is correctly captured by the new model with a near-constant behaviour across the entire range of compositions. Conversely, failure of the previous model is highlighted in a C_2_H_4_-poor range (inlet fraction <0.2 mol mol^−1^, dotted red line), where the faulty near-equilibrium assumption for C_2_H_4_ adsorption imposes a linear proportionality of type: STY_C_2_H_6__ = *C*·*x*_C_2_H_4_,in_, resulting in a null *y*-intercept. The new model purposefully drops this hypothesis, avoiding an underestimation of up to 100% of the C_2_H_6_ selectivity, and 20% of the overall reaction heat generated by the exothermal reactions. On a side note, Fig. 7 also shows that the near-equilibrium approximation is justifiable when C_2_H_4_-rich regimes are investigated (*i.e.* “pure” tail-end conditions), as visible from the STY_C_2_H_6__ and STY_C_2_H_6__ trends in the right section of the broken plots.

**Fig. 8:**
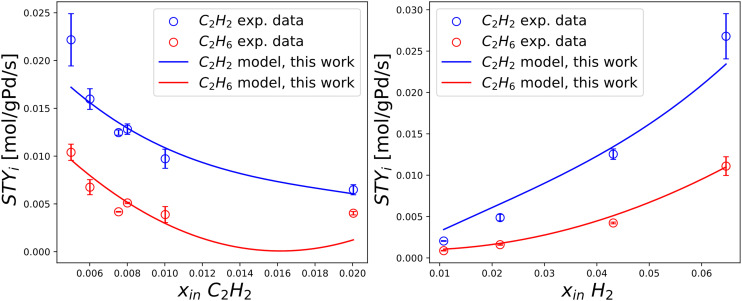
Experimentally measured and modelled space–time yields of C_2_H_2_ and C_2_H_6_ (STY_C_2_H_2__ [mol g_Pd_^−1^ s^−1^] and STY_C_2_H_6__ [mol g_Pd_^−1^ s^−1^]) plotted against single manipulated operating variables. Includes experimental data by Urmes *et al.* and model simulation results by this work. Left) Trends *vs.* inlet molar fraction of C_2_H_2_ obtained at 323 K, *i.e.* 50 °C. Right) Trends *vs.* inlet molar fraction of H_2_ obtained at 323 K, *i.e.* 50 °C. The standard deviation of experimental measurements is reported through error bars for points whose reproducibility has been verified through at least two repeated experiments.

In Fig. 8, the performance of the new model is investigated with variable C_2_H_2_ and H_2_ inlet molar fractions, at a fixed isothermal reactor temperature of 323 K. The experimental data used for this comparison is taken from Urmès *et al.*^34^ according to considerations made in the beginning of this section. Owing to no registered improvement (nor downgrade) to the prior model, only the results of the new model are shown in Fig. 8. Overall, solid evidence is provided on the agreement of the model with experiments, justifying the use of the same model in real post-plasma representative conditions, (section 3.2.2).

#### 3.2.2 Model results in undiluted post-plasma hydrogenation conditions

**Fig. 9:**
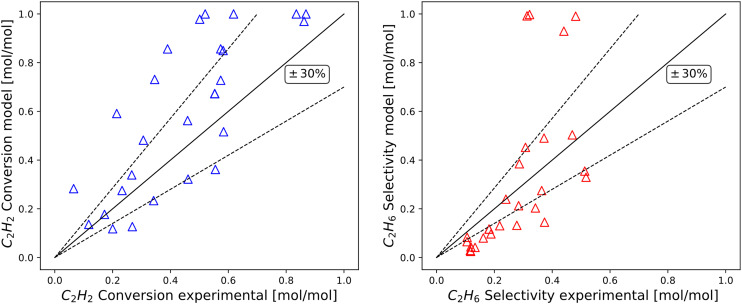
Parity plot for C_2_H_2_ hydrogenation prediction on a undiluted hydrocarbon stream with ±30% relative deviation lines. The *y*-axis corresponds to the results of the computational model and *x*-axis follows experimentally obtained points. Left) C_2_H_2_ conversion right) C_2_H_6_ selectivity.

**Fig. 10:**
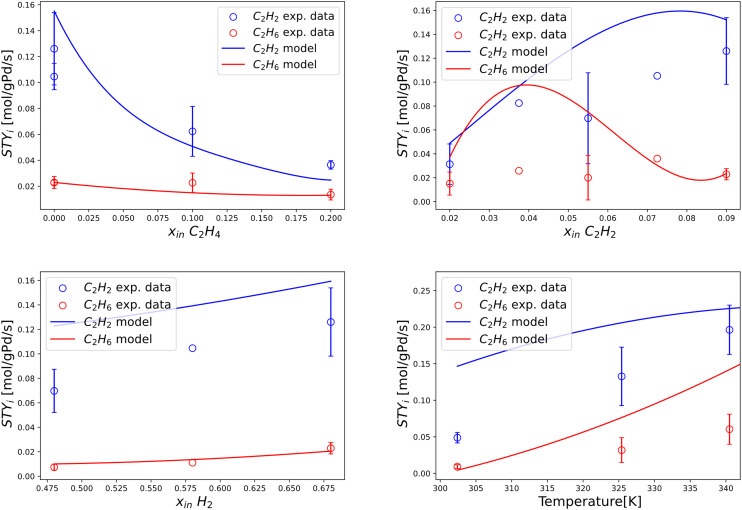
Experimentally measured and modelled space–time yields of C_2_H_2_ consumption and C_2_H_6_ production (STY_C_2_H_2__ [mol g_Pd_^−1^ s^−1^] and STY_C_2_H_6__ [mol g_Pd_^−1^ s^−1^]) plotted against single manipulated operating variables. Top left) Trends *vs.* inlet molar fraction of C_2_H_4_ obtained at 293 K, *i.e.* 20 °C. Top right) Trends *vs.* inlet molar fraction of C_2_H_2_ obtained at 293 K, *i.e.* 20 °C. Bottom left) Trends *vs.* inlet molar fraction of H_2_ obtained at 293 K, *i.e.* 20 °C. Bottom right) Trends *vs.* isothermal reactor temperature. Includes experimental data from this work and model simulation results by this work. The standard deviation of experimental measurements is reported through error bars for points whose reproducibility has been verified through at least two repeated experiments.

This section examines the behaviour and accuracy of the new model in undiluted post-plasma conditions. Similarly to section 3.2.1, the overall performance of the model is shown in terms of parity for C_2_H_2_ conversion and C_2_H_6_ selectivity, reported in Fig. 9 with 30% dispersion lines. Reasonable agreement is observed, in line with the expectations of the challenging exothermal conditions adopted. The wide error bars observed for multiple experimental points in undiluted conditions (Fig. 5) confirm that a certain level of inaccuracy is intrinsic to the undiluted conditions adopted. Nonetheless, the model is generally accurate inside the post-plasma zone of interest (*e.g.*, at high C_2_H_2_ concentration), albeit with a higher observed deviation for C_2_H_6_ selectivity. The resulting trends are analysed in detail within Fig. 10, where continuous lines are adopted to help elucidate the predictive power of the modelled variables.

In Fig. 10, experimentally measured and modelled space–time yields are plotted against the inlet molar fractions of key components and temperature. In Fig. 10 (top left), a decreasing trend is predicted for STY_C_2_H_2__*vs.* inlet molar fraction of C_2_H_4_, in alignment with experiments and diluted trends of Fig. 7. A near-constant value of STY_C_2_H_6__ is highlighted for the same plotted trend, in agreement with experiments. Great accuracy is shown at an inlet molar fraction of C_2_H_4_ = 0, which is the most important condition in terms of post-plasma application (see Table 1). Such prediction is non-trivial since it is qualitatively incompatible with the previous model of Urmès,^11^ where a null inlet molar fraction of C_2_H_4_ would necessarily lead to null predicted value of STY_C_2_H_6__ as shown in 3.2.1 (Fig. 7).

In Fig. 10 (top right), reasonable experimental agreement is observed for the modelled trend of STY_C_2_H_2__*vs.* inlet molar fraction of C_2_H_2_ with an increased accuracy towards high C_2_H_2_ content (*i.e.* post-plasma conditions). A maximum is observed for the predicted trend at inlet molar fraction of C_2_H_2_ = 0.08 mol mol^−1^, which is in general agreement with experimental evidence and compatible – after the maximum – with the behaviour reported for a diluted case in Fig. 7 (*i.e.* decreasing STY_C_2_H_2__ with increasing C_2_H_2_ amount). In turn, the general trend of the modelled STY_C_2_H_6__*vs.* inlet molar fraction of C_2_H_2_ seems to agree with experiments -albeit with relevant deviations in the absolute value-, and provide a similar trend to Fig. 7 after the maximum. It is hard to ascertain whether the location of the maximum predicted by the model agrees with experiments owing to the large error bars of the investigated points. Overall, it is plausible that the maxima for STY_C_2_H_2__ and STY_C_2_H_6__*vs.* inlet molar fraction of C_2_H_2_ for the two cases (undiluted and diluted) lie at different molar fractions, and are outside of the experimental ranges investigated in the diluted case. For more definitive evidence, further refinement should be adopted in diluted conditions, beyond the scope of this work. More details on the kinetic analysis are found in SI (section S3).

The correct prediction of both STY_C_2_H_2__ and STY_C_2_H_6__ trends *vs.* inlet molar fraction of H_2_ is demonstrated in Fig. 10 (bottom left). A strict increase of STY in relation to H_2_ content is observed, as expected of a hydrogenation reaction whose H_2_ reaction order is typically larger than 1.

Finally, Fig. 10 (bottom right) shows reasonable accuracy of the model at different post-plasma operational temperatures, from 300 K to 340 K, with an overestimation of C_2_H_6_ production.

### 3.3 Comparison with literature and model consistency

Fitted values of the parameters are compared with both recent experimental evidence and theoretically calculated values for C_2_H_2_ hydrogenation on a Pd surface. Owing to the presence of grouped constants, *i.e. A*_4_, *A*_7_, *E*_a4_, *E*_a7_, *n*_1_, *n*_2_,^28,30,43^ the focus of this analysis is placed on the adsorption equilibrium constants *K*_1_, *K*_2_, *K*_5_.

Starting from Table 3, the similarities and discrepancies in the fitted adsorption equilibrium constants are highlighted. The regressed values of *K*_1_ and *K*_5_ (for H_2_ and C_2_H_4_, respectively) are in relatively close agreement between diluted and undiluted conditions. They are also in vicinity of regressed values from literature, with a maximum factor 10–50 observed deviation. The strongest difference is observed for the C_2_H_2_ adsorption equilibrium constant (*K*_2_), with a nearly factor 100 difference between diluted and undiluted conditions. Multiple factors can cause this. First, this model neglects the effect of coverage, which would affect C_2_H_2_ the most (see literature^19^). Second, the difference in metallic Pd loading between the catalysts used in undiluted and diluted conditions (1 wt% Pd *vs.* 0.05 wt%, respectively, Table 2) can lead to strong deviations in the fitted kinetic parameters, although not thermodynamic in nature. In particular, metal loading can affect the arrangement of Pd atoms and Pd–H phases, affecting the hydrogenation activity of the catalyst significantly.^15,44–47^ Last, errors in the other fitted parameters (*i.e. A*_4_, *A*_7_, *E*_a4_, *E*_a7_, *n*_1_, *n*_2_) inherently affect the predicted adsorption equilibrium constants, deeming the observed maximum value of deviation (10^2^) acceptable.

Finally, the consistency of adsorption equilibrium parameters is analysed on a theoretical basis starting from *ab initio* reported values of chemisorption free energy Δ*G*_ads_ or chemisorption enthalpy Δ*H*_ads_. Tabulated values of free energy variation are used where available, allowing direct calculation of adsorption equilibrium constants, while adsorption enthalpy values are used where free energy is not provided (see SI, section S7).

Starting from *K*_2_, the only instance of chemisorption free energy calculated at high coverage for C_2_H_2_ in literature^19^ leads to an estimated value of *K*_2_ (10^2^ kPa^−1^ at 300 K) in relatively good agreement with the values from this work in a similar temperature range (10^3^–10^5^ kPa^−1^, Table 3). Hereby, the midpoint of the highest adsorbate-adsorbate interaction, *i.e.* the self-interaction of C_2_H_2_ Δ*G*_ads,C_2_H_2__(*θ*_C_2_H_2__), has been used in the comparison, with only qualitative validity in the wide instantaneous coverage range predicted in most of this work's conditions (*e.g. θ*_C_2_H_2__ = 0.1–0.7, *θ*_C_2_H_4__ = 0.2–0.9, *θ*_H_ = 0.1–0.4, θ_*_ = 10^–5^–10^−3^). Other theoretical studies have confirmed a marked increase in C_2_H_2_ chemisorption free energy, going from strongly negative values to gradually smaller ones, although never exploring a coverage regime above 0.5 ML,^48,49^*i.e.* this work's postulated conditions.

Concerning C_2_H_4_, the di-σ mode of adsorption is deemed compatible with the fitted value of *K*_5_, out of the two main adsorption modes of C_2_H_4_ (di-σ and -π) postulated to occur on a Pd surface.^25,47,50,51^ These two modes have a respective heat of adsorption of −70 and −39 kJ mol^−1^, and the former leads to a calculated value of *K*_5_ (10^3^–10^4^ kPa^−1^ at 300 K) in close agreement with the *K*_5_ constant fitted values in this work in a similar temperature range (10^2^–10^3^ kPa^−1^).^25,47,50,51^ The non-negligible production of C_2_H_6_ observed in all conditions hints at a prevalence of stronger C-Pd bonding at high coverage *via* di-σ adsorption, which π-adsorption would fail to explain despite being theoretically favoured at higher coverage.^25,47,50,51^

At last, the value of *K*_1_ fitted to experiments (10^3^–10^4^ kPa^−1^) is in general agreement with the H_2_ adsorption equilibrium value calculated with transition state theory assuming dissociative adsorption accounting for self-interactions^40^ (10^4^–10^5^ kPa^−1^ at 300 K). Overall, the agreement of the fitted adsorption parameters with relevant literature is good, providing further evidence of the model's validity.

## 4. Conclusions

In this study, a new kinetic model is developed for Pd-based hydrogenation of C_2_H_2_ in post-plasma conditions. The assumption of reversible C_2_H_4_ adsorption is introduced to describe a wide range of experimental conditions, as testified by the agreement of the model with the extended tail-end data provided by the authors. The shortcomings of traditional LHHW models are highlighted and surpassed, and the new model is applied to previously undisclosed post-plasma conditions. Herein, highly exothermal runs with an adiabatic rise >100 K are investigated and shown to agree with the model, confirming model accuracy in conditions that have never been studied. Future work by the authors will explore the integration of this model within a multi-dimensional reactor simulation framework, exploiting the validity of computationally light kinetic predictions for thermal optimization purposes in existing, heuristic post-plasma reactor instances (see ref. 4 and 5). Finally, the scope of model application should be extended beyond plasma reactors, to any instance where a C_2_-rich/C_2_H_2_-rich hydrocarbon stream can be valorized using a hydrogenation reaction.

## Author contributions

Victor Rosa: conceptualization, methodology, investigation, writing – original draft, writing – review & editing. Fabio Cameli: conceptualization, investigation, writing – review & editing. Yves Schuurman: conceptualization, writing – review & editing. Kevin M. Van Geem: conceptualization, writing – review & editing. Georgios D. Stefanidis: conceptualization, writing – review & editing.

## Conflicts of interest

There are no conflicts to declare.

## Supplementary Material

CY-015-D5CY00529A-s001

## Data Availability

The data supporting this article have been included as part of the SI. Supplementary information is available. See DOI: https://doi.org/10.1039/D5CY00529A.
