# Supplementary material for: A kinetic model for Pd-based hydrogenation of acetylene-rich streams typical of post-plasma applications
Source: Catal Sci Technol. 2025 Sep 3;15(23):7014–29. doi: 10.1039/d5cy00529a (PMC12442368; doi:10.1039/d5cy00529a)
Supplement: CY-015-D5CY00529A-s001 [file CY-015-D5CY00529A-s001.pdf]

Supplementary Information (SI) for Catalysis Science & Technology.
This journal is © The Royal Society of Chemistry 2025

ARTICLE

A kinetic model for Pd-based hydrogenation of acetylene-rich streams typical of post-plasma applications

Victor Rosa, [a] Fabio Cameli, [b] Yves Schuurman, [c] Kevin M. Van Geem,*[a] Georgios Stefanidis,*[a,b]

Supporting information

S1.Kinetic model details

S1.1 Derivation

The derivation of the model presented in this work follows the simplified reaction scheme from Fig. S1, left (also reported in the paper's Fig. 3), using a single active Pd site hypothesis, where adsorbed species are indicated with a * subscript. It is hereby assumed that reactivity occurs on a single layer of adsorbates (θ_{i*}), i.e., a monolayer, and that the quasi-stationary state approximation can be adopted for surface intermediates (QSSA, see for instance Boudart[1]). Accordingly, several of the reactive stages, including steps 1,2,3, and 6, are considered near-equilibrium (i.e., in quasi-equilibrium, QE). Concurrently, steps 4 and 7 are assumed to be the two irreversible rate-determining steps (RDS), which drive the overall consumption rate of acetylene (C_2H_2) and production rate of ethane (C_2H_6), respectively. The typical closure of this system with the QSSA approach would imply that step 5 is also at equilibrium, similarly to previous instances of the same mechanism in the literature[2, 3]. Conversely, the adsorption of C_2H_4 (step 5) is hereby assumed to be neither rate-limiting nor near-equilibrium, but rather a fully reversible step. Referring to the well-known concept of affinity described by Boudart[1], this implies that step 5 has neither zero nor unitary affinity, differing from the typical scenario of QSSA application. The step does not determine the overall consumption or production rate of any specific molecule (i.e. single path), but intervenes in the closure of the system through the adsorbed C_2H_4 surface balance $\left(\frac{\partial \theta_{C_2H_4*}}{\partial t}\right)$, as will be shown later in this section. In this way, the first part of the mechanism (steps 1-3) and the second part (steps 6,7) are connected and consistent with the typical QSSA methodology. This proposed approach is intuitively described in Fig. S1, where a graphical comparison outlines the differences of the latter with existing QSSA models from literature[3]. Hereby, the reversibility of step 5 is highlighted, and its value obtained as a summation of the first and the second RDS (while the relative lengths of arrows are not intended to be accurate) is clearly shown. The choices of QE and RDS used in both models of Fig. S1 for the comparison (this model and the old QSSA) are supported by prior evidence and methodology cited in the main body of work. Similarly, the additional choice of reversibility for C_2H_4 adsorption (step 5) proposed in this new model is motivated by the failure of previous models in post-plasma representative conditions, as illustrated in the main body of work (Fig. 6).

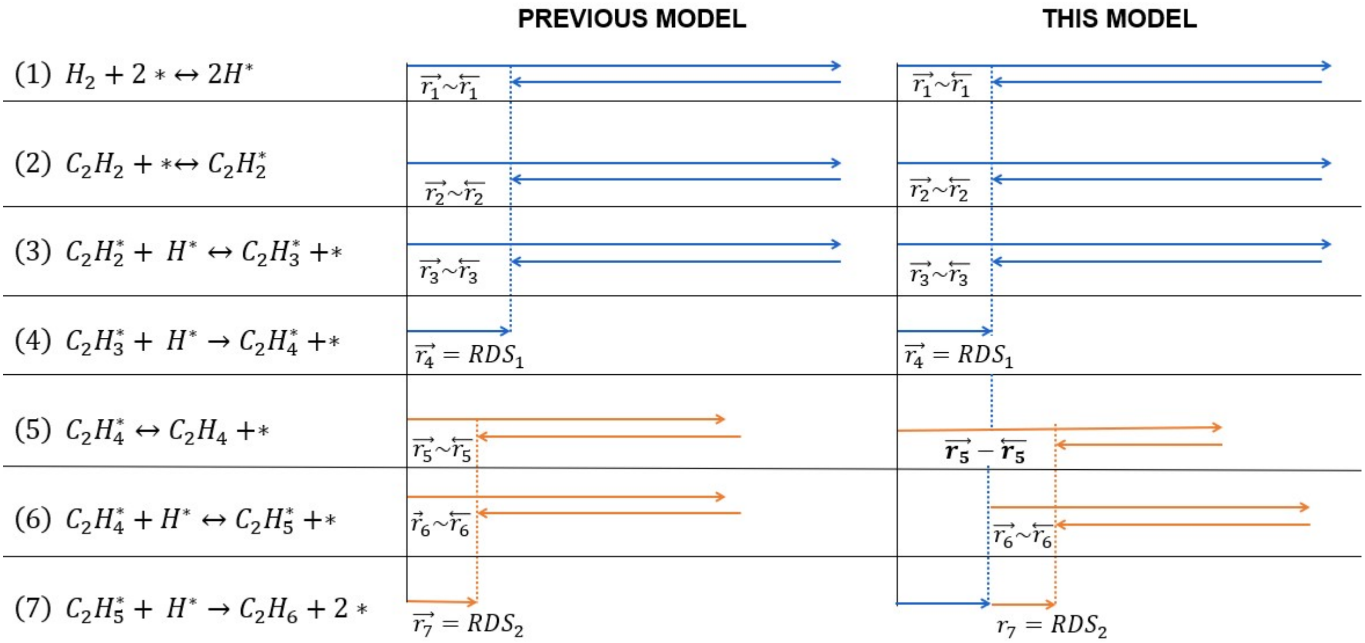

Figure S1 *Illustration of the hybrid QSSA approach hereby proposed for the hydrogenation of C_2H_2 in post-plasma representative conditions. The numbering of steps refers to Fig. 3 in the main body of work. The use of the quasi-equilibrium (QE) and of the rate-determining step (RDS) assumptions is highlighted, as well as the reversibility of step 5.*

The considerations so far made can be translated into mathematical form through Eqs. ~~1~~S1-6. These provide an analytical relationship between the coverage of adsorbed acetylene, hydrogen, vinyl, ethyl ($\theta_{C_2H_2^*}, \theta_{H^*}, \theta_{C_2H_3^*}, \theta_{C_2H_5^*}$) and reactant partial pressures (p_i), including free site coverage (θ_*). Specifically, the use of the QE approximation for steps 1,2,3,6 leads to Eqs. ~~1~~S1,2,3,5. The RDS assumption for steps 4 and 7 leads to Eq. ~~4~~S4 and ~~6~~S6.

$$\vec{r}_1 \sim \overleftarrow{r}_1 \rightarrow \vec{k}_1 p_{H_2} \theta_*^2 = \overleftarrow{k}_1 (\theta_{H^*})^2 \tag{S1}$$

$$\vec{r}_2 \sim \overleftarrow{r}_2 \rightarrow \vec{k}_2 p_{C_2H_2} \theta_* = \overleftarrow{k}_2 \theta_{C_2H_2^*} \tag{S2}$$

$$\vec{r}_3 \sim \overleftarrow{r}_3 \rightarrow \vec{k}_3 \theta_{C_2H_2^*} \theta_{H^*} = \overleftarrow{k}_3 \theta_{C_2H_3^*} \theta_* \tag{S3}$$

$$\vec{r}_{4,RDS\,1} = -r_{C_2H_2} = \vec{k}_4 \theta_{C_2H_3^*} \theta_{H^*} \tag{S4}$$

$$\vec{r}_6 \sim \overleftarrow{r}_6 \rightarrow \vec{k}_6 \theta_{C_2H_4^*} \theta_{H^*} = \overleftarrow{k}_6 \theta_{C_2H_5^*} \theta_* \tag{S5}$$

$$\vec{r}_{7,RDS\,2} = r_{C_2H_6} = \vec{k}_7 \theta_{C_2H_5^*} \theta_{H^*} \tag{S6}$$

This journal is © The Royal Society of Chemistry 20xx

Please do not adjust margins

Please do not adjust margins

Using equilibrium relations of type $K_{eq,i}=\frac{\vec{k}_i}{\overleftarrow{k}_i}$ (shown in Eq.7 of the paper), Eqs. ~~1~~S1-6 can be rearranged after basic substitutions into expressions of type: $\theta_i=f(p_i,\theta_*)$ shown in Eqs ~~7~~S7-12, which have a similar form to prior work for the same mechanism[3].

$$\theta_{H*}=\left(K_1p_{H_2}\right)^{0.5}\theta_* \tag{S7}$$

$$\theta_{C_2H_2*}=\left(K_2p_{C_2H_2}\right)\theta_* \tag{S8}$$

$$\theta_{C_2H_3^*}=K_3\frac{\theta_{C_2H_2^*}\theta_{H^*}}{\theta_*}=\left(K_3K_2K_1^{0.5}\right)p_{C_2H_2}p_{H_2}^{0.5}\theta_* \tag{S9}$$

$$r_{4,\overrightarrow{RDS}1}=r_{C_2H_2}=(\vec{k}_4K_3K_2K_1)\,p_{C_2H_2}p_{H_2}\;\theta_*^2 \tag{S10}$$

$$\theta_{C_2H_5*}=K_6\frac{\theta_{C_2H_4^*}\theta_{H^*}}{\theta_*} \tag{S11}$$

$$r_{7,\overrightarrow{RDS}2}=r_{C_2H_6}=(\vec{k}_7K_6\frac{\theta_{C_2H_4^*}\theta_{H^*}^2}{\theta_*}) \tag{S12}$$

In the resulting system of equations, all coverages are explicitly defined, except for $\theta_{C_2H_4^*}$. As anticipated, the system is closed through a $\theta_{C_2H_4^*}$ surface balance. The terms accounting for consumption/production of $\theta_{C_2H_4^*}$ via Scheme 1 are $r_{4,\overrightarrow{RDS}}$, $r_{5,\overrightarrow{ads}}$, $\overleftarrow{r}_6$, $r_{5,\overleftarrow{des}}$ and $\overleftarrow{r}_6$. The use of QSSA within this work's methodology implies a steady-state nature of this system. Therefore, Eq. ~~13~~S13 can be written for stationary $\theta_{C_2H_4^*}$ coverage, i.e. $\frac{\partial\theta_{C_2H_4^*}}{\partial t}=0$, with a typically microkinetic modelling approach[4]. This marks the hybrid character of the model presented with respect to a standard Langmuir-Hinshelwood (LH) model.

$$\frac{\partial\theta_{C_2H_4^*}}{\partial t}\left[\frac{mol}{mol_{Pd}\cdot s}\right]=^{S.S.}0=r_{5,\overleftarrow{ads}}-r_{5,\overrightarrow{des}}+r_{4,\overrightarrow{RDS}}-\vec{r}_6+\overleftarrow{r}_6 \tag{S13}$$

By analysing the terms appearing in Eq. ~~13~~S13 and looking at Scheme 1, it is clear that the consumption/production of $\theta_{C_2H_4*}$ via step 6 (i.e. term: $-\vec{r}_6+\overleftarrow{r}_6$) is necessarily equal to its consumption via step 7 ($-r_{7,\overrightarrow{RDS}2}$) with the assumptions adopted. On a strictly formal mass-balance basis, this is inconsistent due to the QE assumption for step 6 ($\vec{r}_6\sim\overleftarrow{r}_6\rightarrow\vec{r}_6-\overleftarrow{r}_6\sim0$), but similarly to the QSSA approach, it allows to obtain an inexpensive analytical solution of the system which can be a good approximation of the complex numerical one. Eq. ~~13~~S13 can thus be rewritten as Eq. ~~14~~S14.

Please do not adjust margins

$$\frac{\partial \theta_{C_2H_4^*}}{\partial t} \overset{S.S.}{=} 0 = \overleftarrow{r}_{5,ads} - \overrightarrow{r}_{5,des} + \overrightarrow{r}_{4,RDS} - \overrightarrow{r}_{7,RDS} \qquad \text{(S14)}$$

A further simplification is employed at this stage. The extensive experimental evidence in this work suggests that production rate of C_2H_6 only accounts for a small fraction of the C_2H_2 consumption rate below full C_2H_2 conversion, around 10-20%, i.e. $\overrightarrow{r}_{4,RDS} \gg \overrightarrow{r}_{7,RDS}$. The value of $\overrightarrow{r}_{7,RDS}$ -which accounts for C_2H_6 production $r_{C_2H_6}$ as seen in Eq. ~~12~~S12- is relatively constant across the various reacting conditions investigated. Therefore, a near-constant 20% over-prediction $\theta_{C_2H_4^*}$ can be expected if $\overrightarrow{r}_{7,RDS}$ is neglected in Eq. ~~14~~S14. This is within the experimental error of the campaigns, as well as the 95% confidence intervals of the parameters fitted, and simplifies the analytical solution procedure substantially (ultimately leading to quadratic equation rather than a cubic one in Eq. ~~20~~S20). Therefore, it is used as an assumption of choice, retaining the practicality in this model's adoption. Eq. ~~14~~S14 can be rewritten as Eq. ~~15~~S15, where the assumed dependence of $\overrightarrow{r}_{5,ads}$ and $\overrightarrow{r}_{5,des}$ on $p_{C_2H_4}$, $\theta_{C_2H_4^*}$, θ_* $\overrightarrow{k}_5$ and $\overleftarrow{k}_5$ are made explicit.

$$0 = \overleftarrow{r}_{5,ads} + \overleftarrow{r}_{5,des} + \overrightarrow{r}_{4,RDS} = \overrightarrow{k}_5 p_{C_2H_4}\theta_* - \overleftarrow{k}_5\theta_{C_2H_4^*} + \overrightarrow{k}_4 K_3K_2K_1\, p_{C_2H_2}p_{H_2}\theta_*^2 \qquad \text{(S15)}$$

Eq. ~~15~~S15 can be then rearranged to provide an explicit dependence of $\theta_{C_2H_4}$ on partial pressures and free site coverage, in Eq. ~~16~~S16.

$$\theta_{C_2H_4^*} = K_5 p_{C_2H_4}\theta_* + \frac{\overrightarrow{k}_4 K_3K_2K_1)\, p_{C_2H_2}p_{H_2}\theta_*^2}{\overleftarrow{k}_5} \qquad \text{(S16)}$$

With the relationship above, the dependence of $\theta_{C_2H_5}$ on partial pressures and free site coverage can also be explicated, by substituting Eq. ~~16~~S16 into Eq. ~~11~~S11 (yielding Eq. ~~17~~S17).

$$\theta_{C_2H_5^*} = K_6\left(K_1 p_{H_2}\right)^{0.5}\left(K_5 p_{C_2H_4}\theta_* + \frac{\overrightarrow{k}_4 K_3K_2K_1\, p_{C_2H_2}p_{H_2}\theta_*^2}{\overleftarrow{k}_{5,des}}\right) = K_6K_5K_1^{0.5}p_{C_2H_4}p_{H_2}^{0.5}\theta_* + \left(\frac{\overrightarrow{k}_4 K_6K_3K_2K_1^{1.5}\, p_{C_2H_2}p_{H_2}^{1.5}}{\overleftarrow{k}_{5,des}}\right)\theta_*^2 \qquad \text{(S17)}$$

Finally, by substituting Eq. ~~17~~S17 into Eq. ~~12~~S12, an expression of type $f(p_i, \theta_*)$ is obtained for $\overrightarrow{r}_{7,RDS}$, shown in Eq. ~~18~~S18.

$$r_7 = RDS_2 \rightarrow \ \overrightarrow{k}_7\,\theta_{C_2H_5^*}\,\theta_{H^*} = \overrightarrow{k}_7\,\theta_{C_2H_5^*}\left(K_1 p_{H_2}\right)^{0.5}\theta_* =$$

$$\vec{k_7} K_6\left[K_5K_1 p_{C_2H_4}p_{H_2}\theta_*^2 + \left(\frac{\vec{k_4} K_3K_2K_1^2 p_{C_2H_2}p_{H_2}^2}{\overleftarrow{k}_{5,des}}\right)\theta_*^3\right] \qquad (S18)$$

The last unsolved variable above is the free site coverage θ_*. Similarly to standard QSSA methodology, the latter can be obtained by a surface site balance, where the sum of adsorbates needs to amount to unity, as written in Eq. ~~19~~S19. Hereby, of all the adsorbates, only the most-abundant reactive intermediates (MARI) $\theta_{C_2H_2^*}, \theta_{H^*}, \theta_{C_2H_4^*}$ are accounted for, owing to their expected larger coverage in this work's condition and prior evidence in this regard[2,3]. The functional form of the expressions derived is only slightly affected by neglection of $\theta_{C_2H_3^*}, \theta_{C_2H_5^*}$, leading to minimal effect on fitness quality and a decrease in variable correlation (see discussion after Eq. ~~20~~S20).

$$1 = \theta_{C_2H_4^*} + \theta_{C_2H_2^*} + \theta_{H^*} + \theta_* = \frac{\vec{k_4} K_3K_2K_1 p_{C_2H_2}p_{H_2}}{\overleftarrow{k_5}}\theta_*^2 + \left[K_2p_{C_2H_2} + K_5p_{C_2H_4} + \left(K_1p_{H_2}\right)^{0.5} + 1\right]\theta_* \qquad (S19)$$

Eq. ~~19~~S19 is a second-order algebraic equation with a positive root and a negative one, with a positive, definite Δ. The positive solution is the only one that has physical meaning, leading to Eq. ~~20~~S20

$$a\theta_*^2 + b\theta_* - 1 = 0 \rightarrow \theta_* = \frac{-b + \sqrt{b^2 - 4ac}}{2a} \qquad (S20)$$

$$where \quad a = \frac{\vec{k_4} K_3K_2K_1 p_{C_2H_2}p_{H_2}}{\overleftarrow{k_5}},\ b = \left[K_2p_{C_2H_2} + K_5p_{C_2H_4} + \left(K_1p_{H_2}\right)^{0.5} + 1\right],\ c = -1.$$

The neglection of $\theta_{C_2H_3^*}, \theta_{C_2H_5^*}$ coverages allows to reduce parameter correlation by means of two grouped constants k_4, k_7 (Eq. ~~21~~S21-22) that are fitted against experimental data with modified Arrhenius-type expressions. In this way, the equilibrium parameters K_3, K_6 never appear isolated and are grouped together with $\vec{k_4}, \vec{k_7}$, within the two final fitted constants k_4, k_7 as shown in Eq. ~~21~~S21-22. Therein, the dependence of activation energies on coverages is neglected owing to lack of sufficient data in the literature, and the multiplication properties of exponentials are exploited to obtain the final expressions. In particular, a modified Arrhenius relation is used, owing to the strong temperature dependences that can arise in the $\frac{\vec{A_3}\,\vec{A_4}}{A_3}, \frac{\vec{A_6}\,\vec{A_7}}{A_6}$ groups, which formally depend on the activation entropies of single steps $\Delta S_i^{\neq}$ according to transition-state theory.[5, 6]

Please do not adjust margins

$$k_4 = \vec{k}_4 K_3 = \vec{k}_4 \frac{\vec{k}_3}{\overleftarrow{k}_3} = \frac{\left(\vec{A}_3\, e^{-\frac{\vec{E}_{a3}}{RT}}\right)\vec{A}_4\, e^{-\frac{\vec{E}_{a4}}{RT}}}{\overleftarrow{A}_3 e^{-\frac{\overleftarrow{E}_{a3}}{RT}}} = \frac{\vec{A}_3\,\vec{A}_4}{A_3} e^{-\frac{\vec{E}_{a3}+\vec{E}_{a4}-\overleftarrow{E}_{a3}}{RT}} = A_4 T^{n_4} e^{-\frac{E_{a4}}{RT}} \tag{S21}$$

$$k_7 = \vec{k}_7 K_6 = \vec{k}_7 \frac{\vec{k}_6}{\overleftarrow{k}_6} = \frac{\left(\vec{A}_6\, e^{-\frac{\vec{E}_{a6}}{RT}}\right)\vec{A}_7\, e^{-\frac{\vec{E}_{a7}}{RT}}}{\overleftarrow{A}_6 e^{-\frac{\overleftarrow{E}_{a6}}{RT}}} = \frac{\vec{A}_6\,\vec{A}_7}{A_6} e^{-\frac{\vec{E}_{a6}+\vec{E}_{a7}-\overleftarrow{E}_{a6}}{RT}} = A_7 T^{n_7} e^{-\frac{E_{a7}}{RT}} \tag{S22}$$

The grouped constants k_4,k_7 are thus substituted in Eq. ~~18~~S18 and ~~20~~S20 and the deriving variables A_4,n_4,E_{a4},A_7,n_7,E_{a7} are used for the fitting purposes of this work.

S2. Statistical models and fitness quality

Hereby, additional analysis is performed on the results presented in the main body of work, including derivation of the confidence interval calculation formula and statistical correlation based on the Jacobian of the residual vector. Based on **an extension** of linear regression statistics to non-linear cases[7], the Jacobian matrix for a regression problem is calculated as follows:

$$J = \frac{\partial\left(y_{i,model} - y_{i,exp}\right)}{\beta_i} \tag{S24}$$

Where β_i are the fitted parameters, $y_{i,model}$ represent the modelled variable (i.e. integral reaction rates calculated with Eq. ~~9~~S9 in the main body of work) and $y_{i,exp}$ are the single experimental points (i.e. space-time yields calculated with Eq. 1 from main body of work or space-time consumption rates calculated with adaptation thereof). This matrix is calculated by the least_squares package in Python, through finite differences and a linear loss method. For the fitted parameters β_i, an approximate 1-α marginal confidence interval can be calculated with the following relationship[7]:

$$\beta = \overline{\beta} \pm \frac{\left[\sum_i^N \left(y_{i,model} - y_{i,exp}\right)^2\right]}{N-P}\sqrt{\left(J^T J\right)}\ t\left(N-P,\frac{\alpha}{2}\right) = (Var - covar\ matrix)\cdot\ t\left(N-P,\frac{\alpha}{2}\right) \tag{S25}$$

Please do not adjust margins

Please do not adjust margins

Where N is the number of responses (i.e. experimental points) and P is the number of parameters, t is the student t-distribution value calculated with the latter and the grouped matrix $\frac{\left[\sum_i^N (y_{i,model} - y_{i,exp})^2\right]}{N-P}\sqrt{(J^T J)}$ is called the variance-covariance matrix. Furthermore, the binary correlation parameters are calculated as shown in Eq. ~~26~~S26.

$$\rho_{i,j} = \frac{(Var - covar\ matrix)_{i,j}}{\sqrt{(Var - covar\ matrix)_{i,i}\,(Var - covar\ matrix)_{j,j}}} \tag{S26}$$

The parameter correlation matrix for the diluted ~~case~~and undiluted cases of interest is reported in Figure S2.

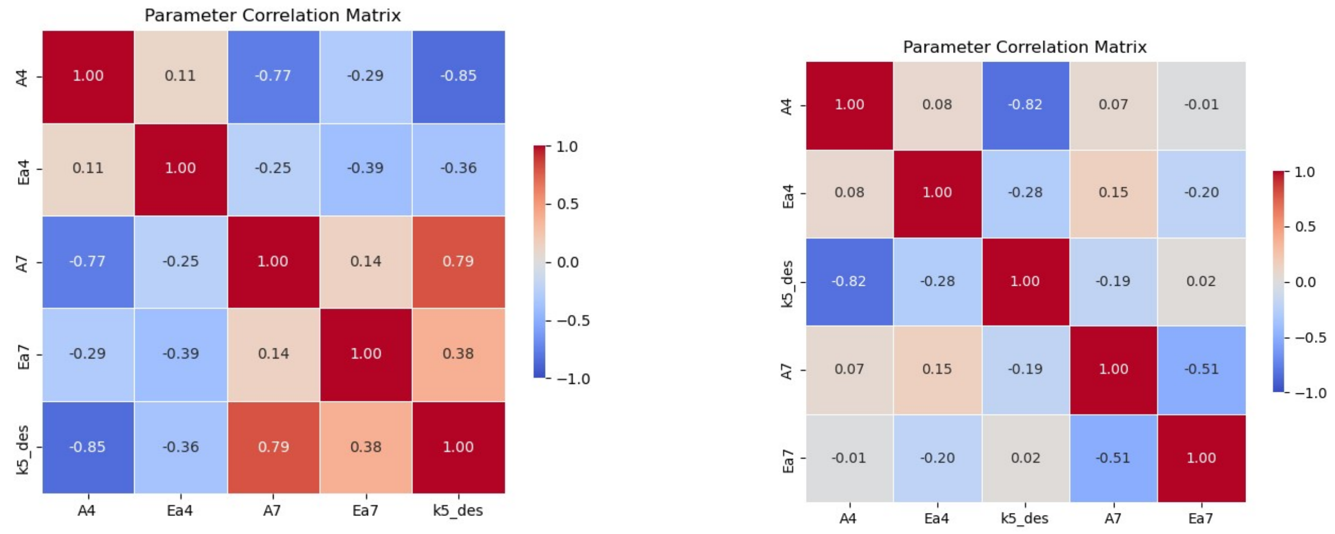

***Figure S2** Left) Parameter correlation matrix for the model fitted on experimental diluted hydrocarbon stream data, with a color-based representation of correlation. Right) Parameter correlation matrix for the model fitted on experimental undiluted hydrocarbon stream data, with a color-based representation of correlation.*

In the undiluted case, the correlations between A_4 and $k_{5,des}$, A_7 and $k_{5,des}$ are the strongest ones, still below the limit 0.9 value which is usually used as threshold for unacceptable correlation. In the undiluted case, the correlation between A_4 and A_7 is at the limit of correlation tolerance. These are deemed reasonable in the limited temperature range (and data range) used for fitting. Furthermore, the presence of grouped terms $\frac{k_4}{k_{5,des}}$, $\frac{k_7}{k_{5,des}}$ appearing in both $r_{C_2H_2}$, $r_{C_2H_6}$ and the site balance in Eqs. S19-20 explains part of the correlation observed. Only in one instance do A_4 and $k_{5,des}$ appear separate, i.e. in $r_{C_2H_6}$calculation, and the same is true for A_7 and $k_{5,des}$. The limited isolated appearance of these constants is the reason underlying their separate fitting, and their strong correlation. Furthermore, $r_{C_2H_6}$ is lower than $r_{C_2H_2}$ and has likely lower weight in the variance-covariance matrix, further

Please do not adjust margins

corroborating the explanation behind the strong $A_4 \leftrightarrow k_{5,des}$ correlation. The resulting confidence intervals for both the diluted and undiluted case are relatively small, indicating that the fitness of the model is adequate.

In principle, the fitting should be repeated with an increased number of experimental points. This could be achieved either by performing new experiments on already verified behaviors or by using combined, transient downstream analytical techniques, such as mass spectroscopy (MS). If the number of points is sufficient (e.g., see ref.[8]), statistical analysis can be performed effectively on a much larger number of parameters as typically done in microkinetic analyses. At the same time, such approaches place less emphasis on the reproducibility of the data, which is one of the highlights of this work. The lack of existing data in the literature on part of the regimes investigated provides a strong rationale for this choice.

S3~~.~~ Partial pressure~~, temperature and residence-time~~ dependence

Following the presentation of model results section 3.2.2 in the main body of work, the implications of the kinetic rates adopted in this work are hereby analysed. In particular, the maximum behaviour observed in an undiluted case for the C_2H_6 production (at an inlet fraction of C_2H_2=0.~~05~~07, Fig. 5 main text, ~~Top Right~~top right) is in agreement with the hypothesis of a rate-determining step subsequent to C_2H_2 adsorption (in our case, step ~~4) and this will be hereby proven. Every reaction rate~~4The analysis presented in ~~the paper -and consequence of the latter hereby extrapolated~~ this section is based on the hypothesis that the integral mean of reaction rates is a relatively close ~~representation~~approximation of their respective instantaneous rates~~, i.e.~~ $\int$ ~~from Eq. 9 in the paper.~~ (non-integral). This may ~~not be true in all cases, but is especially~~ be reasonable ~~in the~~at limited- conversion[9] ~~regimes investigated hereby.~~. Nonetheless, it will be shown that the trends observed and modelled agree with this assumption. Recalling Eq. S20, the solution of free active site coverage is calculated as shown in Eq-~~26-27~~S27-28.

$$a\theta_l^2 + b\theta_l - 1 = 0 \tag{S27}$$

$$\theta_l = \frac{-b + \sqrt{b^2 - 4ac}}{2a} \quad where\ a = \left(\frac{k_4 K_2 K_1 p_{C_2H_2} p_{H_2}}{k_{5,des}}\right)\theta_l^2, \tag{S28}$$

$$b = (K_5 p_{C_2H_4} + \left(K_1 p_{H_2}\right)^{0.5} + K_2 p_{C_2H_2} + 1, \qquad c = -1$$

The implications of Eq.S27 are analysed through superposition of effects in two extreme cases, namely when $a \gg b$ and $a \ll b$. This is shown in Eqs. ~~26-30~~S29-33, where the functional expressions obtained for the reaction rates in the two extreme cases are shown.

$$if\ a \gg b,\ \ \theta_l = \frac{1}{\sqrt{a}} = \frac{1}{\sqrt{\frac{k_4 K_2 K_1 p_{C_2H_2} p_{H_2}}{k_{5,des}}}} \qquad if\ a \ll b,\ \ \theta_l = \frac{1}{b} = \frac{1}{1 + (K_5 p_{C_2H_4} + \left(K_1 p_{H_2}\right)^{0.5} + K_2 p_{C_2H_2}} \tag{S29}$$

$$if\ a \gg b,\ \ r_{C_2H_2} \sim k_4K_2K_1p_{C_2H_2}p_{H_2}\theta_l^2 = \frac{k_4K_2K_1p_{C_2H_2}p_{H_2}}{\frac{k_4K_2K_1p_{C_2H_2}p_{H_2}}{k_{5,des}}} \quad \text{(S30)}$$

$$r_{C_2H_6} \sim (\frac{k_4\,k_7K_2K_1^2/k_{5,des})\,p_{C_2H_2}p_{H_2}^2}{\left(\frac{k_4K_2K_1p_{C_2H_2}p_{H_2}}{k_{5,des}}\right)^{1.5}} + \frac{k_7K_5K_1p_{C_2H_4}p_{H_2}}{\frac{k_4K_2K_1p_{C_2H_2}p_{H_2}}{k_{5,des}}} \quad \text{(S31)}$$

$$if\ a \ll b,\ \ r_{C_2H_2} \sim k_4K_2K_1p_{C_2H_2}p_{H_2}\theta_l^2 = \frac{k_4K_2K_1p_{C_2H_2}p_{H_2}}{\left(1 + \ (K_5p_{C_2H_4} + \left(K_1p_{H_2}\right)^{0.5}\ + K_2p_{C_2H_2}\right)^2} \quad \text{(S32)}$$

$$r_{C_2H_6} \sim (\frac{k_4\,k_7K_2K_1^2/k_{5,des})\,p_{C_2H_2}p_{H_2}^2}{\left(1 + (K_5p_{C_2H_4} + \left(K_1p_{H_2}\right)^{0.5}\ + K_2p_{C_2H_2}\right)^3} + \frac{k_7K_5K_1p_{C_2H_4}p_{H_2}}{\left(1 + (K_5p_{C_2H_4} + \left(K_1p_{H_2}\right)^{0.5}\ + K_2p_{C_2H_2}\right)^2} \quad \text{(S33)}$$

The assumption of $a \gg b$ yields the expressions in Eqs. ~~30,31~~S30,S31 which seemingly disagrees with the predictions shown in the main body of text (Fig. 5). Conversely, the assumption of $a \ll b$ yields Eq. S32,33 which agree with the trend observed. Both the $\frac{x}{(C + K_2x)^2}$ trend predicted for $r_{C_2H_2}\ vs.\ p_{C_2H_2}$ and the $\frac{x}{(C + K_2x)^3}$ trend predicted for $r_{C_2H_6}\ vs.\ p_{C_2H_2}$ are curves with a maximum and an asymptotic constant behavior after the maximum. Both trends seem compatible with the experimental observations, where the existence of a maximum of $r_{C_2H_6}$ can be postulated -within experimental error- from Fig. 5 in the main body of work, and the maximum of $r_{C_2H_2}$ can be assumed outside of the range of experiments, as specified in the main body of work. Since two extreme cases were hereby taken, the reality will lie in between the $a \gg b$ and $a \ll b$ assumptions. Conversely, assuming H_2 adsorption as rate-determining would lead to expressions in contrast with experimental evidence. For instance $r_{C_2H_2}$ would be calculated as in Eq.S34, with a strictly negative reaction order for C_2H_2 on $r_{C_2H_2}$.

$$r_{C_2H_2} = K_1p_{H_2}\theta_l^2 \quad \text{(S34)}$$

S4.Catalyst characterization

Please do not adjust margins

Metal dispersion and active surface area are calculated via H_2 chemisorption in a Micromeritics AutoChem 2920 via Eq. S35. Pulsed injections of H_2 interact solely with Pd and not the Al-based support. Therefore, the values reported in

Table ~~S2~~S1 are representative of the effective Pd metal dispersion within the catalyst.

Table S~~2~~1. *Metal dispersion and active surface area of Pd on α-Al_2O_3 for various catalysts*

Catalyst	Pd 0.05 wt.%	Pd 1 wt.%
**Metal dispersion (%)**	~~38~~ ± 2%%	10 ± 2%
**Mass of catalyst used**	100	10
**Mass of effective available Pd [mg]**	0.02	0.01

$$\%Dispersion = \frac{2 \cdot n_{Pd,\, theoretical}}{n_{H_2,\, adsorbed}} = \frac{\frac{2 \cdot m_{cat} \cdot wt.\%_{Pd}}{MW_{Pd}} \cdot 100}{n_{H_2\, fed\, single\, pulse} \cdot \frac{\left(\frac{\sum_{i}^{N-k} A_{H_2}^{i+k}}{N-k} - A_{H_2}^{i=1} \right)}{\left(\frac{\sum_{i+1}^{N} A_{H_2}^{i}}{N-1} \right)}} \tag{S35}$$

Where m_{cat} is the mass of catalyst adopted, $wt.\%_{Pd}$ is the weight percentage of Pd in the catalyst, MW_{Pd} is the molecular weight of Pd, $n_{H_2\, fed\, single\, pulse}$ is the amount of H_2 fed in a single pulse measured in moles and $A_{H_2}^{i}$ is the area of H_2 measured via thermal conductivity detector (TCD) after the i-th pulse.

S5 Experimental procedures

S5.1 Calculation of space-time yields

The experimentally observed variables, i.e. $STY_{C_2H_2}$ and $STY_{C_2H_6}$ are calculated as shown in Eq. 1 in the main body of work. In the latter, the outlet molar flow rates in mol s^{-1} are missing. At each reacting condition, several GC injections are performed to ensure the presence of steady-state conditions. When these are reached, the molar flow rates are calculated with the following procedure using CH_4 as internal standard owing to its verified inert behaviour (cross-compared with He) and its similar amount in the feed to the target molecules. Firstly, the conversion of C_2H_2 ($\chi_{C_2H_2}$) is calculated as:

Please do not adjust margins

Please do not adjust margins

$$\chi_{C_2H_2} = \frac{\left(A_{GC_{C_2H_2}}^{in} - A_{GC_{C_2H_2}}^{out} \frac{A_{GC_{CH_4}}^{in}}{A_{GC_{CH_4}}^{out}}\right)}{A_{GC_{C_2H_2}}^{in}} \quad (S36)$$

Where $A_{GC_{C_2H_2}}^{in}, A_{GC_{CH_4}}^{in}$ are the calibrated GC peak areas of C_2H_2 and CH_4 in the inlet stream, and $A_{GC_{C_2H_2}}^{out}, A_{GC_{CH_4}}^{out}$ are the outlet peak areas of C_2H_2 and CH_4 detected in the steady-state conditions. The outlet molar flow rate of C_2H_2, $F_{C_2H_2}^{out}$, can then be calculated as:

$$F_{C_2H_2}^{out} = F_{C_2H_2}^{in} - F_{C_2H_2}^{in} \cdot \chi_{C_2H_2} \quad (S37)$$

Where $F_{C_2H_2}^{in}$ is the known inlet molar flow rate of C_2H_2 fed to the reactor, calculated as:

$$F_{C_2H_2}^{in}\left[\frac{mol}{s}\right] = \frac{\left(V^{in}\left[\frac{NmL}{min}\right] \cdot x_{C_2H_2}^{in}\right)}{22.4\frac{L}{mol} \cdot 60\left[\frac{s}{min}\right] \cdot 1000\left[\frac{L}{mL}\right]} \quad (S38)$$

Where V^{in} is the inlet volumetric flowrate of the mixture and $x_{C_2H_2}^{in}$ is the volumetric fraction of C_2H_2 in the inlet mixture, both controlled via the mass-flow controllers of the feed section of the setup.

The outlet ~~flow rate~~flowrate of C_2H_6 , $F_{C_2H_6}^{out}$, is calculated through C_2H_6 selectivity ($\sigma_{C_2H_6}$), defined as:

$$\sigma_{C_2H_6} = \left[\frac{A_{GC_{C_2H_6}}^{out} - A_{GC_{C_2H_6}}^{in}}{\left(A_{GC_{C_2H_2}}^{in} \cdot \chi_{C_2H_2}\right)}\right] \cdot GC_{fac,C_2H_6} = \frac{A_{GC_{C_2H_6}}^{out} - A_{GC_{C_2H_6}}^{in}}{\left(A_{GC_{C_2H_2}}^{in} \cdot \chi_{C_2H_2}\right)} \cdot 1.23 \quad (S39)$$

Where $A_{GC_{C_2H_6}}^{out}$ and $A_{GC_{C_2H_6}}^{in}$ are the outlet and inlet peak GC areas of C_2H_6, and GC_{fac,C_2H_6} is the GC factor used to relate the C_2H_6 areas to volumetric concentration, using the conversion of C_2H_2 as reference, for which this relation is known and calibrated each time. Finally, the outlet molar flow rate of C_2H_6 is calculated as:

$$F_{C_2H_6}^{out} = F_{C_2H_2}^{in} \cdot \chi_{C_2H_2} \cdot \sigma_{C_2H_6} \quad (S40)$$

S5.2 Carbon mass balance

Please do not adjust margins

To provide an effective measure of mass balance closure, a calculation of carbon lack percentage(%) is performed using C_2H_2 as a reference compound for the calculation of the GC (gas chromatography) conversion factors for the various feed components, adopting CH_4 as internal standard:

$$\% C.lack = \frac{F_{in}\left(\sum_{i}^{N.\,Reactants} N_{C\,atoms,i} A_{GC_i}^{in} G.C._{fac,i}\right) - \frac{F_{in} A_{GC_{CH_4}}^{in}}{A_{GC_{CH_4}}^{out}}\left(\sum_{i}^{N.\,Reactants} NC_i A_{GC_i}^{out} GC_{fac,i}\right)}{F_{in}\left(\sum_{i}^{N.\,Reactants} NC_i A_{GC_i}^{in} GC_{fac,i}\right)} \quad (S41)$$

$$GC_{fac,i} = \frac{\left(\frac{x_i^{in}}{A_{GC_i}^{in}}\right)}{\left(\frac{x_{C_2H_2}^{in}}{A_{GC_{C_2H_2}}^{in}}\right)} \quad (S42)$$

Where $N_{C\,atoms,i}$ is the equivalent number of carbon atoms for the molecule i, $\frac{F_{in} A_{GC_{CH_4}}^{in}}{A_{GC_{CH_4}}^{out}}$ is the effective outlet flowrate considering the effect of reaction on the total volumetric/molar flowrate by using CH_4 as inert internal standard (IS). The GC factor for molecules absent in the feed stream is calculated by either prior calibration (C_2H_6, 1.23 factor) or theoretical consideration (C_4, factor 2 proportional to the number of carbon atoms in the molecule). CH_4 is considered inert in all conditions investigated, based on prior evidence that volumetric flow changes calculated with CH_4 and a He IS are near-identical. At the same time, the incomplete closure of balances at times can be attributed to minor effects of CH_4 reactivity, to the relative error on the assumed correction factors used for species that are absent in the feed, as well as undetected (aromatic) byproduct formation[10].[9]. Therefore, the effect of accounting for C_4 products detected by the GC columns, such as C_4H_{10} (butane), 2-C_4H_8 (E/Z butene),1-C_4H_8 (butene), 2-C_4H_8 (E/Z butene) and 1-C_4H_6 (butyne) has been tested, with minimal differences. The results of % C lack calculation are shown in Table S3 and S4. As in prior instances[10]As in prior instances[9], C_4 products (and subsequent green-oil formation) are the main relevant byproducts generated by the coupling reactions of the C_2 reactants fed to the reactor. Heavier species tend to condense under the form of polyaromatic hydrocarbons (PAH), which are observed on the surface of the catalyst bed after several hour of operation (with no evident effect on reactivity). The time-on-stream of the trials performed has never surpassed 48h, therefore minimizing the likelihood of effects related to coke-deposition and catalyst deactivation[11][10], and making a proper assessment of the latter beyond the scope of the work presented.

Table S32 *%C lack for most relevant points of the experimental set gathered in diluted tail-end conditions (referring to section 2 of the main article), where inlet% of C_2H_2 is fixed at 0.75%, inlet %of CH_4 is fixed at 1.9%, %H_2 is fixed at 4.3%.*

Please do not adjust margins

Temp.[C]	Mol/mol% inlet $\%C_2H_4$	%C lack (no C4)	%C lack (including C4)
51	0.4%	9%	6%
51	0.4%	7%	5%
51	10.0%	1%	1%
51	15.0%	4%	4%
51	20.0%	1%	1%
51	30.0%	3%	3%
51	30.0%	2%	2%
61	0.4%	13%	9%
61	0.4%	9%	6%
61	30.0%	2%	2%
61	30.0%	1%	1%

Table S43 *%C lack for most relevant points of the experimental set gathered in undiluted post-plasma conditions (referring to section 2 of the main article)*

Temp.[C]	Mol/mol% inlet						
	$\%C_2H_4$	$\%C_2H_2$	$\%H_2$	$\%CH_4$	%Ar	%C lack (no C4)	%C lack (including C4)
20	0%	9%	58%	23%	10%	19%	13%
19	0%	9%	48%	23%	20%	13%	11%
30	0%	5%	24%	12%	60%	9%	6%
23	0%	9%	68%	23%	0%	9%	7%
20	0%	9%	68%	23%	0%	5%	4%
20	0%	9%	68%	23%	0%	3%	3%
15	10%	7%	54%	18%	10%	7%	5%
40	10%	7%	54%	18%	10%	8%	5%
25	20%	7%	54%	18%	0%	5%	3%
18	0%	6%	68%	23%	4%	10%	9%
40	0%	6%	68%	23%	4%	10%	6%
24	0%	9%	68%	23%	0%	8%	5%

This journal is © The Royal Society of Chemistry 20xx

Please do not adjust margins

Please do not adjust margins

S5.3. Data reproducibility

The experimental data presented throughout the paper is provided with error bars calculated on the basis of their standard deviation. In particular, the definition of standard deviation for a generic experimental condition and the respective measured reaction rates space-time yield is shown in Eq. 46S43.

$$dev.st.() = \frac{\sum \Theta \sum \Theta}{} \tag{S43}$$

Where N is the number of repeated experiments (at least 2) and _is the calculated average of the reaction ratesspace-time yields for the specific point investigated. The procedure to calculate has already been shown in S6.1 with reference to the main body of work.

S6.Reactor model equation

Under the hypothesis of an isothermal pseudo-homogeneous packed bed reactor with no intraparticle or gas-phase diffusional limitations, the following continuity equation is adopted for species conservation on a mass basis:

$$m_{tot}\frac{\partial Y_i}{\partial z} = \sum_{j=0}^{N.R.} v_{i,j} r_j \left[\frac{kmol}{m_{Pd} s}\right] \frac{m_{Pd,reactor}}{V_{solids}} MW_i \left[\frac{kg}{kmol}\right] (1-\epsilon)\, A_{sectional,reactor} \tag{S44}$$

Where m_{tot} is the mass flow rate entering the reactor in $\left[\frac{kg}{s}\right]$, Y_i is the mass fraction [-] of the i-th species at a given length in the reactor, $\sum_{j=0}^{N.R.} v_{i,j} r_j []$ is the molar production/consumption rate of the i-th species in $\left[\frac{kmol}{g_{Pd} s}\right]$, $m_{Pd,reactor}$ is the available active mass of Pd metal in the reactor in $[g]$ as measured from chemisorption, V_{solids} is the volume of solid catalyst in the reactor$[m^3]$, ϵ is the porosity of the reactor bed[-], and $A_{sectional,reactor}$ is the cross sectional area of the reactor in $[m^2]$.

S7. Theoretical details

S7.1 Adsorption equilibrium computation based on adsorption enthalpies

The calculation of adsorption equilibrium constants for comparative purposes is performed as per Eq. 44S45.

Please do not adjust margins

$$K_{eq,i}[Pa^{-1}] = \frac{\vec{k}_i}{\overleftarrow{k}_i} \quad (S45)$$

Where $\vec{k}_i$ is the adsorption rate coefficient for a generic adsorption reaction, and $\overleftarrow{k}_i$ is the respective desorption rate coefficient**.** Transition state theory's formulation for surface reactions[1, 4] is used therein. All adsorption processes are assumed to have a tight transition state[5, ~~12~~11], with a negligible activation energy, yielding the temperature-dependent expression for an adsorption rate coefficient shown in Eq. ~~45~~S46[12, 13, ~~14~~]:

$$k_{ads,i}\left[\frac{1}{Pa\cdot s}\right] = \frac{\left(\frac{k_B T}{2\pi m_i}\right)^{0.5}\sigma_i A_s}{RT} = \frac{\sigma_i A_s}{(2\pi MW_i RT)^{0.5}} \quad (S46)$$

Where σ_i is the sticking probability for the adsorbed molecule, MW_i is the molecular weight of the adsorbed molecule , A_s is the surface area of one mole of accessible Pd sites ~~14~~13 (e.g. 10^4 m^2 mol^{-1}, for Pd (111) crystal configuration). Unitary sticking coefficients are assumed for C_2H_2, C_2H_4 and a 0.5 coefficient is used for H_2.~~15~~ ~~For desorption rate coefficients, Eq. 46~~[14] For desorption rate coefficients, Eq. S47 is used in case of a molecular desorption process (i.e. for C_2H_2 and C_2H_4 with the state-of-the-art theory of Campbell[5]) and Eq. ~~47~~S48 is used for associative desorption reactions (i.e. for H_2). The latter makes use of the assumption that only translational degrees of freedom are affected in the evolution from adsorbate to the transition state in the desorption process.

$$k_{des,i}\left[\frac{1}{s}\right] = \frac{k_B T}{h} e^{\left(\frac{\Delta S^{\neq,des,i}}{R}\right)} e^{\left(\frac{\Delta H_{ads,i}}{RT}\right)} = \frac{k_B T}{h}\left(0.3S^0_{gas,i} + 3.3R - S^{0,}_{gas,i,1D\ trans}\right) e^{\left(\frac{\Delta H_{ads,i}}{RT}\right)} \quad (S47)$$

$$k_{des,i}\left[\frac{mol_{Pd}}{mol\cdot s}\right] = \frac{h}{2\pi\, A_s m_{i^*}} \frac{m^{\neq}_i}{m_{i^*}} e^{\left(\frac{\Delta H_{ads,i}}{RT}\right)} \quad (S48)$$

Where k_B is the Boltzmann constant equal to 1.38 · 10^{-23} $[J\cdot K^{-1}]$, h is the reduced Planck constant equal to 10^{-34} $[J\cdot s]$, S^0_{gas} is the molar entropy of the respective gas-phase molecule at the investigated temperature and pressure in $\left[\frac{J}{mol\cdot K}\right]$, $S^{0,}_{gas,1D\ trans}$ is the 1D-translational entropy of the molecule in the same conditions with same units, $\Delta H_{ads,i}$ is its adsorption enthalpy in $\left[\frac{J}{mol}\right]$, and $m^{\neq}_i, m^*_i$ are the masses of the associative transition state and the adsorbates, respectively, expressed in $[kg]$. The explicit dependence of adsorption enthalpies on coverage is hereby neglected in view of a simplified analysis. Furthermore, owing to a weak dependence of gas-phase entropies on temperature in the range of interest (50K temperature interval), Eq. S47 is used with the tabulated gas-phase entropy values of C_2H_2 and C_2H_4 at 298 K and 1 bar, i.e. 200 and 219 J/mol/K respectively.

Please do not adjust margins

Eq. S47 leads to a grouped $\frac{k_B T}{h}\left(0.3S^{0}_{gas,i} + 3.3R - S^{0,}_{gas,i,1D\ trans}\right)$ value that is approximately 100 times higher than the standard 10^{13} $\left[\frac{1}{s}\right]$ value estimated by TS theory, i.e. 10^{15} $\left[\frac{1}{s}\right]$. Eq. S48 leads to a 10^{13} $[-]$ calculated value for hydrogen associative desorption, in vicinity with the most recent evidence available[15] (i.e.S48 leads to a 10^{13} $[-]$ calculated value for hydrogen associative desorption, in vicinity with the most recent evidence available[14] (i.e. 10^{12} value).

S7.2 Units

For adsorbates appearing throughout this work, a choice is made to use coverages θ_{i^*} expressed in monolayers $\left[\frac{mol}{mol_{Pd}}\right]$, rather than surface concentrations in $\left[\frac{mol}{m^2}\right]$ typical of conventional treatment of transition-state (TS) theory applied to surface reactions.[1, 4] Consistency is always ensured by means of the surface area of Pd $A_{s,Pd}$, assumed equal to 1.26 $10^4\left[\frac{m^2_{Pd}}{mol_{Pd}}\right]$ according to literature [3], which can be used to convert surface concentrations in $\left[\frac{mol}{m^2_{Pd}}\right]$ into coverages expressed in $\left[\frac{mol}{mol_{Pd}}\right]$.

S8 Catalyst activation

The activity of the 1% wt. Pd catalyst over time for the first experimental point in undiluted conditions is shown in Figure S3. As indicated in the main body of work, no activation phase is observed after the H_2 reduction step in the transition towards the sampled points for C_2H_2 experiments.

This journal is © The Royal Society of Chemistry 20xx

Please do not adjust margins

Please do not adjust margins

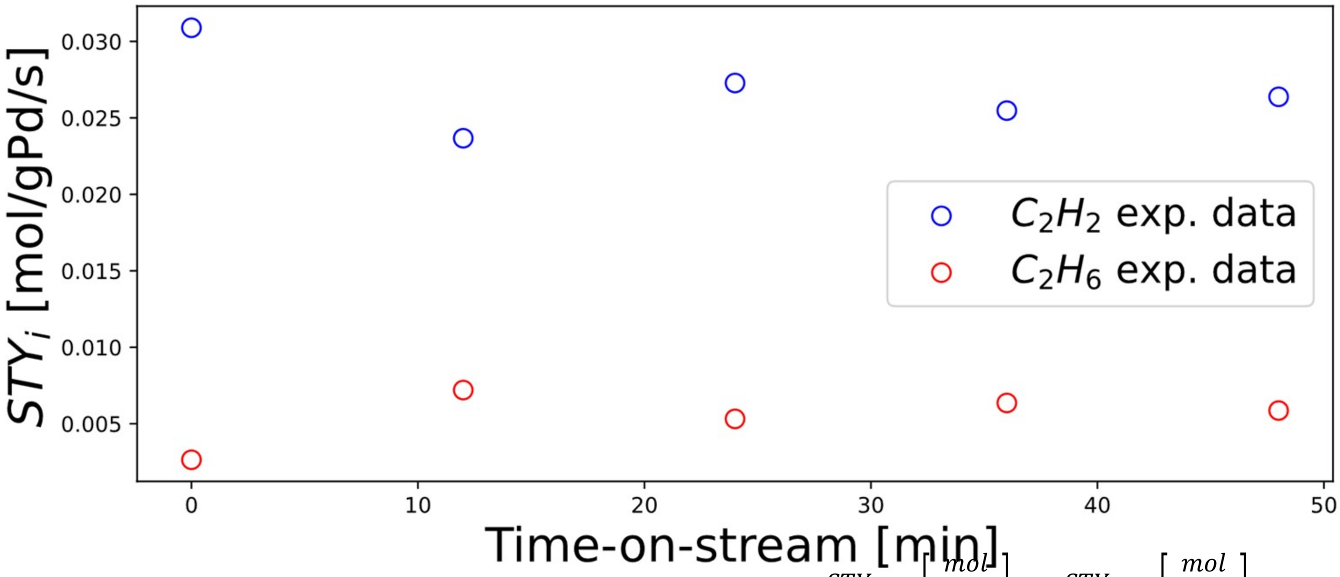

Figure S3 *Experimentally measured space-time yields of C_2H_2 and C_2H_6 (* $STY_{C_2H_2}\left[\frac{mol}{g_{Pd}\cdot s}\right]$ and $STY_{C_2H_6}\left[\frac{mol}{g_{Pd}\cdot s}\right]$ *) vs. the time-on-stream after the H_2 reduction and cooling of the reactor. Trends sampled at a controlled reactor temperature of 293K, i.e. 20 °C. and an inlet molar composition of C_2H_2,H_2,CH_4=0.09,0.68,0.23*

Please do not adjust margins

Please do not adjust margins

Bibliography

1. Boudart, M.; Djéga-Mariadassou, G., KINETICS OF OVERALL REACTIONS. In *Kinetics of Heterogeneous Catalytic Reactions*, Princeton University Press: 1984; pp 77-117.
2. Urmès, C.; Daniel, C.; Schweitzer, J.-M.; Cabiac, A.; Julcour, C.; Schuurman, Y., Microkinetic Modeling of Acetylene Hydrogenation Under Periodic Reactor Operation. *ChemCatChem* **2022,** *14* (8), e202101826.
3. Urmès, C.; Schweitzer, J.-M.; Cabiac, A.; Schuurman, Y. Kinetic Study of the Selective Hydrogenation of Acetylene over Supported Palladium under Tail-End Conditions *Catalysts* [Online], 2019.
4. Kunz, L.; Maier, L.; Tischer, S.; Deutschmann, O., Modeling the Rate of Heterogeneous Reactions. In *Modeling and Simulation of Heterogeneous Catalytic Reactions*, 2011; pp 113-148.
5. Campbell, C.; Arnadottir, L.; Sellers, J., Kinetic Prefactors of Reactions on Solid Surfaces. *Zeitschrift für Physikalische Chemie* **2013,** *227*, 1435–1454.
6. Campbell, C. T.; Sellers, J. R. V., The Entropies of Adsorbed Molecules. *Journal of the American Chemical Society* **2012,** *134* (43), 18109-18115.
7. Practical Considerations in Nonlinear Regression. In *Nonlinear Regression Analysis and Its Applications*, 1988; pp 67-133.
8. Sprung, C.; Kechagiopoulos, P. N.; Thybaut, J. W.; Arstad, B.; Olsbye, U.; Marin, G. B., Microkinetic evaluation of normal and inverse kinetic isotope effects during methane steam reforming to synthesis gas over a Ni/NiAl2O4 model catalyst. *Applied Catalysis A: General* **2015,** *492*, 231-242.
~~9. Maestri, T., Berger, Kapteijn, Moulijn, Overview of requirements for measurement of intrinsic kinetics in and overview of correlations for characteristics of the G-S and L-S fixed-bed reactor, Internal EUROKIN document. 1998.~~
~~10.~~9. Zhang, J.; Sui, Z.; Zhu, Y.-A.; Chen, D.; Zhou, X.; Yuan, W., Composition of the Green Oil in Hydrogenation of Acetylene over a Commercial Pd-Ag/Al 2 O 3 Catalyst. *Chemical Engineering & Technology* **2016,** *39*.
~~11~~10. Su, H.-X.; Jiao, Y.; Shi, J.-G.; Yuan, Z.-W.; Zhang, D.; Wang, X.-P.; Ren, J.; Liu, D.; Gui, J.-Z.; Gao, H.-Y.; Xu, X.-L., Towards the insights into the deactivation behavior of acetylene hydrogenation catalyst. *Petroleum Science* **2024,** *21* (2), 1405-1414.
~~12~~11. Reuter, K., First-Principles Kinetic Monte Carlo Simulations for Heterogeneous Catalysis: Concepts, Status, and Frontiers. In *Modeling and Simulation of Heterogeneous Catalytic Reactions*, 2011; pp 71-111.
~~13~~12. Asnin, L.; Chekryshkin, Y.; Fedorov, A. A., Calculation of the sticking coefficient in the case of the linear adsorption isotherm. *Russian Chemical Bulletin - RUSS CHEM BULL* **2003,** *52*, 2747-2749.
~~14~~13. Sellers, H., Modeling the kinetics of large sets of reactions on metal surfaces. *Russian Journal of Physical Chemistry B* **2007,** *1* (4), 377-393.
~~15~~14. Schwarzer, M.; Hertl, N.; Nitz, F.; Borodin, D.; Fingerhut, J.; Kitsopoulos, T. N.; Wodtke, A. M., Adsorption and Absorption Energies of Hydrogen with Palladium. *The Journal of Physical Chemistry C* **2022,** *126* (34), 14500-14508.

This journal is © The Royal Society of Chemistry 20xx

Please do not adjust margins
